# Necroptosis-Related lncRNAs: Predicting Prognosis and the Distinction between the Cold and Hot Tumors in Gastric Cancer

**DOI:** 10.1155/2021/6718443

**Published:** 2021-11-08

**Authors:** Zirui Zhao, Haohan Liu, Xingyu Zhou, Deliang Fang, Xinde Ou, Jinning Ye, Jianjun Peng, Jianbo Xu

**Affiliations:** Department of Gastrointestinal Surgery, First Affiliated Hospital of Sun Yat-Sen University, Guangzhou 510080, Guangdong Province, China

## Abstract

**Background:**

In the face of poor prognosis and immunotherapy failure of gastric cancer (GC), this project tried to find new potential biomarkers for predicting prognosis and precision medication to ameliorate the situation.

**Methods:**

To form synthetic matrices, we retrieved stomach adenocarcinoma transcriptome data from Genotype-Tissue Expression Project (GTEx) and The Cancer Genome Atlas (TCGA). Necroptosis-related prognostic lncRNA was identified by coexpression analysis and univariate Cox regression. Then we performed the least absolute shrinkage and selection operator (LASSO) to construct the necroptosis-related lncRNA model. Next, the Kaplan–Meier analysis, time-dependent receiver operating characteristics (ROC), univariate Cox (uni-Cox) regression, multivariate Cox (multi-Cox) regression, nomogram, and calibration curves were made to verify and evaluate the model. Gene set enrichment analyses (GSEA), principal component analysis (PCA), immune analysis, and prediction of the half-maximal inhibitory concentration (IC50) in risk groups were also analyzed. For further discussing immunotherapy between the cold and hot tumors, we divided the entire set into two clusters based on necroptosis-related lncRNAs.

**Results:**

We constructed a model with 16 necroptosis-related lncRNAs. In the model, we found the calibration plots showed a good concordance with the prognosis prediction. The area's 1-, 2-, and 3-year OS under the ROC curve (AUC) were 0.726, 0.763, and 0.770, respectively. Risk groups could be a guide of systemic treatment because of significantly different IC50 between risk groups. Above all, clusters could help distinguish between the cold and hot tumors effectively and contribute to precise mediation. Cluster 2 was identified as the hot tumor and more susceptible to immunotherapeutic drugs.

**Conclusion:**

The results of this project supported that necroptosis-related lncRNAs could predict prognosis and help make a distinction between the cold and hot tumors for improving individual therapy in GC.

## 1. Introduction

Regrettably, more often than not, most gastric cancer (GC) patients are diagnosed at an advanced stage with a poor prognosis end [1]. As a result, GC is the third most common cause of cancer death globally (8.2% of 9.6 million cancer deaths in 2018 worldwide) [2]. Systemic treatments are the only choice of patients who cannot be surgically treated. Chemotherapeutics and target therapeutics are common systemic treatments and are frequently reported with treatment failure and side reactions [3]. It urges us to find new exploitable therapeutic strategies. Immunotherapy has had transformed the treatment landscape for malignancies and achieved a lot. But just one-third of patients respond to checkpoint inhibitors in most cancers [4]. Besides failing in the induction of cell death, the cold tumor, lacking preexisting immunity, is also the reason for immunotherapy resistance [5]. Therefore, it is imperative to study how to augment immunotherapy in GC.

As most tumors have innate apoptosis resistance, the induction of other cell death mechanisms, such as necroptosis, has gradually been recognized as promising therapy strategies [4]. Necroptosis, as a novel programmed form of necrotic cell death different from apoptosis, can enhance CD8^+^ leukocyte-mediated antitumor immunity by activating RIPK1 and RIPK3 within the tumor microenvironment (TME) [6]. In another report, a necroptotic cancer cell-mimicry nanovaccine can potentiate antitumor immunity in mice by inducing expansion of NKG2D + natural killer cells and CD8^+^ T cells [4]. At the same time, necroptosis works in generating an immunosuppressive TME to promote malignancies via CXCL1 and Mincle, which also hints at necroptosis as a potential immunotherapy target in GC [7].

Long noncoding RNA (lncRNA) can control genes by influencing their translation or interacting directly with proteins and other RNA species [8]. Linc00176 releases miRNAs, such as miR-9 and miR-185, to downregulate target mRNAs leading to necroptosis of hepatocellular carcinoma cells. TRINGS, the p53-inducible lncRNA, can protect cancer cells from necroptosis by inhibiting TRAP-GSK3*β*-NF-*κ*B necrotic signaling. Cardiomyocyte necroptosis can be regulated by lncRNA through RIPK1/RIPK3 [9]. Besides, lncRNAs have been reported to promote tumors inflammation and help malignancies evade immune destruction [10]. The study of necroptosis-related lncRNA has not been widely mentioned as a potential therapeutic target in GC. Therefore, acquiring more necroptosis-related lncRNAs knowledge can help us understand the roles of necroptosis and lncRNAs in immunotherapy clearly.

The distinction between the cold and hot tumors and turning a cold tumor into a hot tumor will improve the antitumor effects of immunotherapy. It will bring a breakthrough in immunotherapy, while the mechanisms of other cell death remained to be fully elucidated in GC at this stage. But we still lack a simple and effective method for distinguishing tumors [5]. As lncRNAs are spoken highly of acting as new cancer biomarkers in bodily fluids, we tried to regroup patients based on necroptosis-related lncRNAs and identify the hot tumor effectively for improving prognosis and augmenting precise mediation in clinical practice [10, 11].

## 2. Materials and Methods

### 2.1. Acquisition of Information of Patients with GC

For getting synthetic data matrices about stomach adenomas and adenocarcinomas and normal stomach tissue, the RNA transcriptome datasets (HTSeq—Counts and HTSeq—FPKM) and the relevant clinical information were downloaded from Genotype-Tissue Expression Project (GTEx) (https://www.gtexportal.org/) and The Cancer Genome Atlas (TCGA) (https://portal.gdc.cancer.gov/). Then we converted the FPKM value to the TPM value of the synthetic matrix by data.table, tibble, dplyr, and tidyr R packages. As a result, we got two synthetic data matrices. The Counts value matrix was just for identifying differentially expressed lncRNAs, while the TPM value matrix was for the other analyses. To reduce statistical bias in this analysis, stomach adenomas and adenocarcinomas patients with missing overall survival (OS) values or short OS values (<30 days) were excluded. With relevant clinical information, we retrieved 306 patients and divided them into the train risk group and test risk group randomly by Strawberry Perl and caret R package. The ratio was 1:1.

### 2.2. Selection of Necroptosis-Related Genes and lncRNAs

The necroptosis gene set M24779.gmt contains eight necroptosis genes, and it was downloaded from the Gene Set Enrichment Analysis (GSEA) (http://www.gsea-msigdb.org/gsea/index.jsp). In addition, with previous reports about necroptosis, we finally obtained the profile of 67 necroptosis-related genes (Appendix T1). Then we found 5,022 differentially expressed lncRNAs (Log_2_ fold change (FC) > 1, false discovery rate (FDR) < 0.05, and *p* < 0.05) after screening the synthetic data matrix by Strawberry Perl and limma R package [12]. Correlation analysis was performed between 67 necroptosis-related genes and differentially expressed lncRNAs in the combined matrices. Then, 387 lncRNAs, with necroptosis-related genes, Pearson correlation coefficients >0.4, and *p* < 0.001, were considered necroptosis-related lncRNAs.

### 2.3. Establishment and Validation of the Risk Signature

According to the clinical data of GC cases in the TCGA and GTEx, univariate Cox proportional hazard regression analysis was used to screen lncRNAs related to survival from necroptosis-related lncRNA (*p* < 0.05). Then, we made the Lasso regression performed with 10-fold cross-validation and a *p* value of 0.05 as well as run for 1,000 cycles. For each cycle, a random stimulation was set up 1,000 times in order to prevent overfitting. Then a model was established. The 1-, 2-, and 3-year time-dependent receiver operating characteristics (ROC) curves of the model were plotted by the calculation procedure. We calculated the risk score with the following formula:

(1) $$\begin{array}{c} \text{risk score}=\sum_{k=1}^{n}\text{coef}\left({\text{lncRNA}}^{k}\right)*\text{expr}\left({\text{lncRNA}}^{k}\right), \end{array}$$

where the coef (lncRNAn) was the short form of the coefficient of lncRNAs correlated with survival and expr (lncRNAn) was the expression of lncRNAs. According to the median risk score, subgroups including low- and high-risk groups were established [12, 13]. We used the chi-square test to analyze the relationship between the model and clinical factors in order to evaluate the prognostic value of the constructed model.

### 2.4. Independence Factors and ROC

We developed univariate Cox (uni-Cox) and multivariate Cox (multi-Cox) regression analyses to evaluate whether the risk score and clinical characteristics were independent variable factors and made ROC to compare different factors in predicting outcome.

### 2.5. Nomogram and Calibration

With rms R package, the risk score, age, and tumor stage were used to set up a nomogram for the 1-, 2-, and 3-year OS and correction curves based on the Hosmer–Lemeshow test to illustrate whether the prediction outcome showed good consistence with the practical.

### 2.6. Gene Set Enrichment Analyses

With curated gene set (kegg.v7.4.symbols.gmt), gene set enrichment analyses (GSEA) software (https://www.gsea-msigdb.org/gsea/login.jsp) was applied to identify the significantly enriched pathways between the low- and high-risk groups based on the criterion: *p* < 0.05 and FDR < 0.25.

### 2.7. The Investigation of the TME and Immune Checkpoints

According to the result of GSEA, we decided to analyze the immune-cell factors in risk groups. We could calculate the immune infiltration statuses among the GC patients from the TCGA including TIMER, CIBERSORT, XCELL, QUANTISEQ, MCPcounter, EPIC, and CIBERSORT on TIMER2.0 (http://timer.cistrome.org/). In another way, we could download the profile of infiltration estimation for all TCGA tumors on the same website. Wilcoxon signed-rank test, limma, scales, ggplot2, and ggtext R packages were performed in analyzing the differences in immune infiltrating cell content explored, and the results were shown in a bubble chart [13]. Besides, we also made comparisons about TME scores and immune checkpoints activation between low- and high-risk groups by ggpubr R package.

### 2.8. Exploration of the Model in the Clinical Treatment

Then we used the R package pRRophetic to evaluate their therapy response determined by the half-maximal inhibitory concentration (IC50) of each GC patient on Genomics of Drug Sensitivity in Cancer (GDSC) (https://www.cancerrxgene.org/) [14].

### 2.9. Clusters Based on 16 Prognostic lncRNAs

For exploring GC response to immunotherapy, we decided to explore potential molecular subgroups by ConsensusClusterPlus (CC) R package based on the prognostic lncRNAs expression [15]. Principal component analysis (PCA), T-distributed stochastic neighbor embedding (t-SNE), and Kaplan–Meier survival were made by Rtsne R package.

Besides, we made immunity analysis and drug sensitivity comparison by GSVA Base and pRRophetic R package.

## 3. Results

### 3.1. Necroptosis-Related lncRNAs in GC Patients

**Figure 1:**
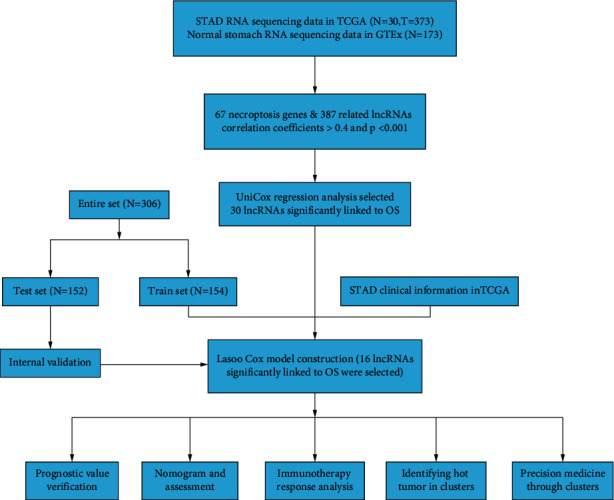
Flow diagram of the study.

**Figure 2:**
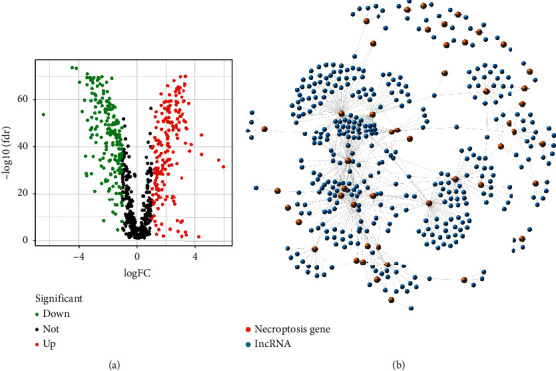
Identification of necroptosis-related lncRNAs in patients with GC. (a) The volcano plot of 387 differentially expressed necroptosis genes. (b) The network between necroptosis genes and lncRNAs (correlation coefficients > 0.4 and *p* < 0.001).

The flow of the study was exhibited in Figure 1. From The Cancer Genome Atlas (TCGA) and Genotype-Tissue Expression Project (GTEx) matrix, we obtained 204 normal samples (174 samples from GTEx) and 343 tumor samples. According to the expression of 67 necroptosis-related genes and differentially expressed lncRNAs (|Log_2_FC| > 1 and *p* < 0.05) between normal and tumor samples, we finally got 387 necroptosis-related lncRNAs (correlation coefficients > 0.4 and *p* < 0.001) [12, 16]. Of them, 194 were upregulated, and the others were downregulated (Figure 2(a)). The network figure and data between necroptosis-related genes, such as AXL and BCL2, and lncRNAs were shown in Figure 2(b) and Appendix D1.

### 3.2. Construction and Verification of the Model

**Figure 3:**
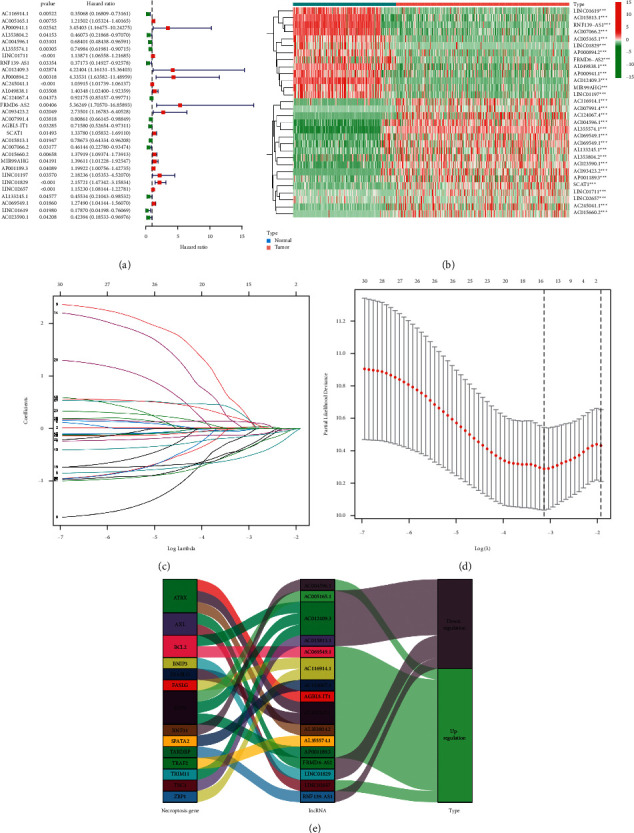
Extraction of necroptosis-related lncRNAs prognostic signature in GC. (a) The prognostic lncRNAs extracted by univariate Cox regression analysis. (b) The expression profiles of 30 prognostic lncRNAs. (c) The 10-fold cross-validation for variable selection in the LASSO model. (d) The LASSO coefficient profile of 16 necroptosis-related lncRNAs. (e) The Sankey diagram of necroptosis genes and related lncRNAs.

According to univariate Cox (uni-Cox) regression analysis, we found 16 necroptosis-related lncRNAs significantly correlated with overall survival (OS) (all *p* < 0.05) and made a heat map (Figures 3(a) and 3(b)). To avoid overfitting the prognostic signature, we performed the Lasso regression on these lncRNAs and extracted 16 lncRNAs related to necroptosis in GC when the first-rank value of Log(*λ*) was the minimum likelihood of deviance (Figures 3(c) and 3(d)). Besides, we could find 10 lncRNAs were upregulated and the others were downregulated in the Sankey diagram (Figure 3(e)).

We calculated risk score with the formula: risk score = LINC01829 × (0.2597) + LINC02657 × (0.1297) + RNF139-AS1 × (−0.1539) + FRMD6-AS2 × (0.0083) + AGBL5-IT1 × (−0.4116) + AC116914.1 × (−0.4053) + AC005165.1 × (0.0228) + AL353804.2 × (−0.0387) + AC004596.1 × (−0.5485) + AL355574.1 × (−0.1209) + AC012409.3 × (0.3441) + AC124067.4 × (−0.0227) + AC015813.1 × (−0.1553) + AP001189.3 × (0.0173) + AL133245.1 × (−0.3709) + AC069549.1 × (0.1690) [13].

**Figure 4:**
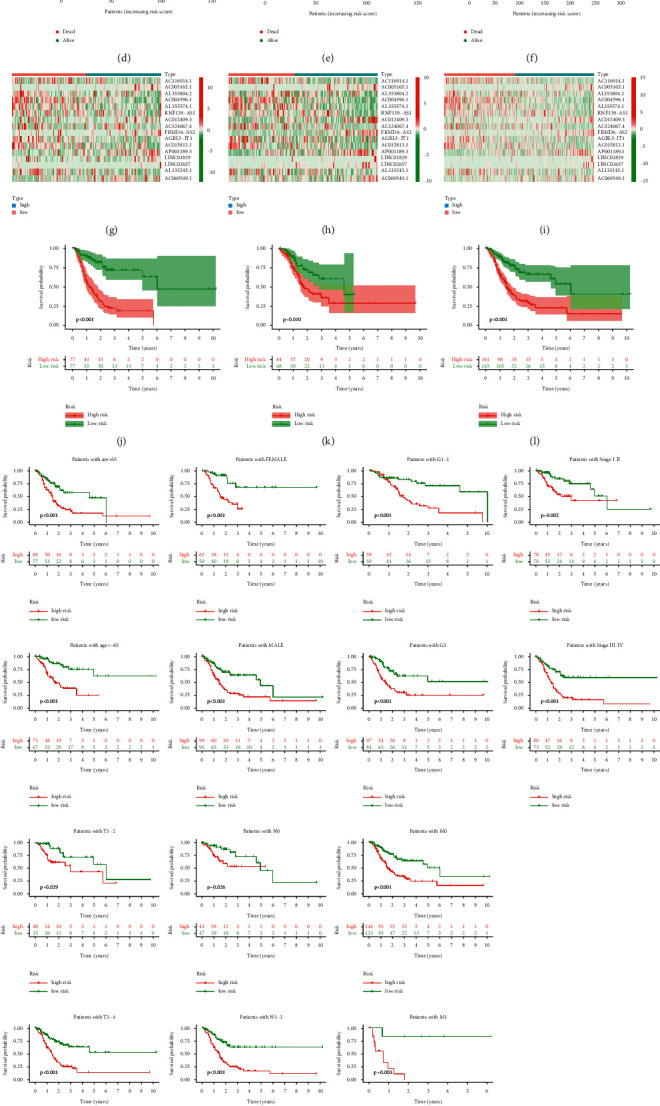
Prognosis value of the 16 necroptosis-related lncRNAs model in the train, test, and entire sets. (a–c) Exhibition of necroptosis-related lncRNAs model based on risk score of the train, test, and entire sets, respectively. (d–f) Survival time and survival status between low- and high-risk groups in the train, test, and entire sets, respectively. (g–i) The heat map of 16 lncRNAs expression in the train, test, and entire sets, respectively. (j–l) Kaplan–Meier survival curves of OS (survival probability) of patients between low- and high-risk groups in the train, test, and entire sets, respectively. (m) Kaplan–Meier survival curves of OS (survival probability) prognostic value stratified by age, gender, grade, stage, T, N, or M between low- and high-risk groups in the entire set.

With the risk score formula, the distribution of risk score, the survival status, survival time, and the relevant expression standards of these lncRNAs of patients were compared between low- and high-risk groups in the train, test, and entire sets. These all indicated the high-risk group had worse prognoses (Figures 4(a)–4(l)). Besides, the conventional clinicopathologic characteristics, age, gender, grade, stage, T, M, and N also performed the same results (Figure 4(m)).

### 3.3. Construction of Nomogram

**Figure 5:**
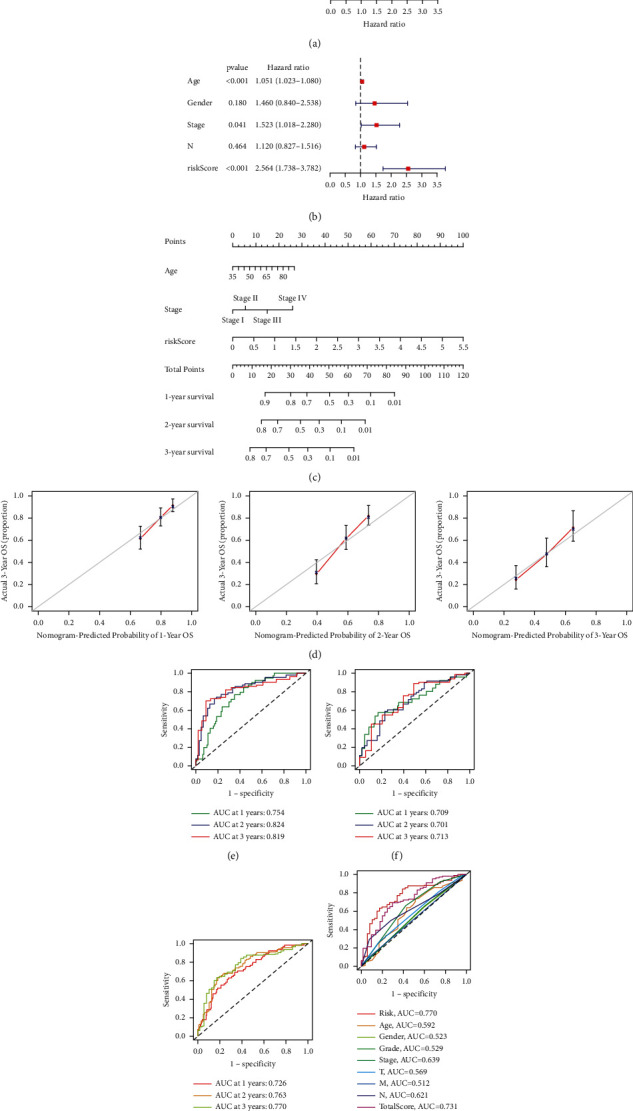
Nomogram and assessment of the risk model. (a, b) Uni- and multi-Cox analyses of clinical factors and risk score with OS. (c) The nomogram that integrated the risk score, age, and tumor stage predicted the probability of the 1-, 2-, and 3-year OS. (d) The calibration curves for 1-, 2-, and 3-year OS. (e–g) The 1-, 2-, and 3-year ROC curves of the train, test, and entire sets, respectively. (h) The 3-year ROC curves of risk score, nomogram total score, and clinical characteristics.

The hazard ratio (HR) of the risk score and 95% confidence interval (CI) were 2.588 and 1.778–3.767 (*p* < 0.001), respectively, in univariate Cox (uni-Cox) regression while 2.564 and 1.738–3.782 (*p* < 0.001), respectively, in multivariate Cox (multi-Cox) regression (Figures 5(a) and 5(b)). In addition, we found the other two independent prognostic parameters, age (1.051 and 1.023–1.080; *p* < 0.001) and stage (1.523 and 1.018–2.280; *p*=0.041) (Figure 5(b)).

According to three independent prognostic factors, risk score, age, and TNM stage (all *p* < 0.05 in multi-Cox), we built a nomogram for predicting the 1-, 2-, and 3-year OS incidences of GC patients (Figure 5(c)). We also utilized the 1-, 2-, and 3-year calibration plots to attest that the nomogram had a good concordance with the prediction of 1-, 2-, and 3-year OS (Figure 5(d)).

### 3.4. Assessment of the Risk Model

Time-dependent receiver operating characteristics (ROC) were utilized to evaluate the sensitivity and specificity of the model on the prognosis. We also illustrated the outcomes of ROC with the area under the ROC curve (AUC). The 1-, 2-, and 3-year AUC of the train set were 0.754, 0.824, and 0.819, of the test set were 0.709, 0.701, and 0.713, and of the entire set were 0.726, 0.763, and 0.770, respectively (Figures 5(e)–5(g)). At the 3-year ROC of risk model, clinical factors and nomogram total score, risk score (0.770), and nomogram (0.731) showed their predominant predictive ability (Figure 5(h)).

### 3.5. GSEA

**Figure 6:**
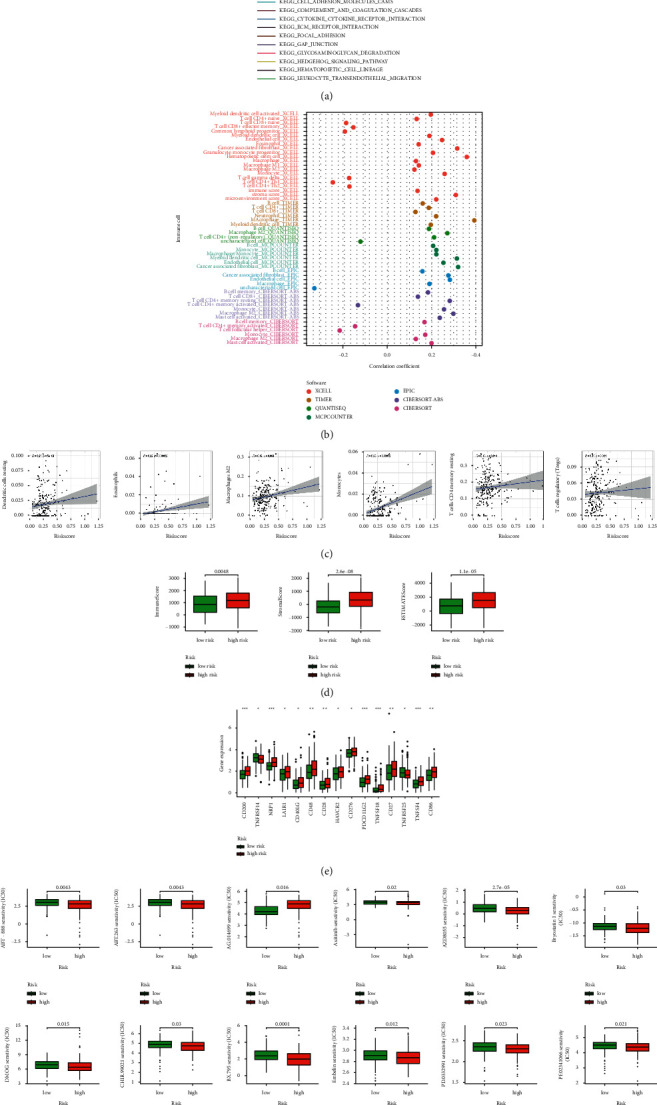
The investigation of tumor immune factors and immunotherapy. (a) GSEA of the top 10 pathways significantly enriched in the high-risk group. (b) The immune cell bubble of risk groups. (c) The correlation between risk score and immune cells. (d) The comparison of immune-related scores between low- and high-risk groups. (e) The difference of 15 checkpoints expression in risk groups. (f) The immunotherapy prediction of risk groups.

To investigate differences in biological functions between risk groups, we utilized GSEA software to explore the high-risk group in the KEGG pathway in the entire set (Figure S1A). Seven of the top ten pathways with enrichment in the high-risk group were highly correlated with tumor invasion, and the others were correlated with immunity such as “leukocyte transendothelial migration” (all *p* < 0.05; FDR < 0.25; |NES| > 1.5) (Figure 6(a)) [17]. Therefore, we tried to make an immunity analysis in the model.

### 3.6. The Investigation of Immunity Factors and Clinical Treatment in Risk Groups

More immune cells were associated with the high-risk group on different platforms exhibited at the immune cell bubble chart and in the document such as macrophage M1, T cell CD4+ naïve, immune score at XCELL, T cell CD8^+^, T cell CD4^+^ at TIMER, T cell CD4^+^ at QUANTISEQ and macrophage at MCPcounter and EPIC (all *p* < 0.05) (Figure 6(b)) (Appendix D2). Besides, we also found that the higher risk score had more association with immune cells such as dendritic cells resting, which had been reported as a part of immunotherapy in GC (Figure 6(c)) [18]. All of these showed the high-risk group had a higher immune infiltration status. The high-risk group had a higher immune score and a higher ESTIMAT (microenvironment) score, signifying a different TME from the low-risk group (Figure 6(d)). Most immune checkpoints also showed better activation in the high-risk group (Figure 6(e)). It implied that we could choose appropriate checkpoint inhibitors for GC patients regrouped by the risk mode [19]. Consistent with reports, the high-risk group, with a higher immune score, had a lower IC50 of 12 immunotherapeutic drugs such as bryostatin 1 (Figure 6(f)) [20]. What's more, we could also find that 16 chemical or targeted drugs, which applied to GC therapy, showed lower IC50 in the high-risk group (Figure S2).

### 3.7. Distinguishing between the Cold and Hot Tumors and Precision Medicine in Clusters

**Figure 7:**
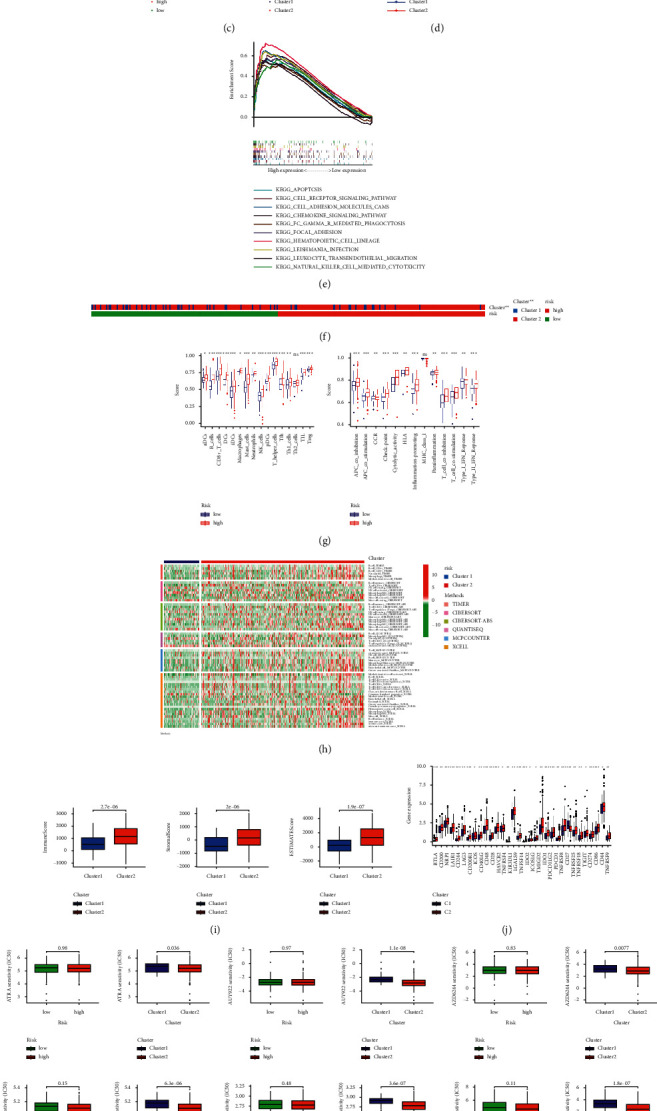
Distinction between cold and hot tumors and immunotherapy prediction. (a) Patients divided into two clusters by ConsensusClusterPlus. (b) The t-SNE of two clusters. (c) The PCA of risk groups and clusters. (d) Kaplan–Meier survival curves of OS in clusters. (e) GSEA of cluster 2. (f) The relationship between risk groups and clusters. (g) The ssGSEA scores of immune cells and immune functions in clusters. (h) The heat map of immune cells in clusters. (i) The comparison of immune-related scores between clusters 1 and 2. (j) The difference of 32 checkpoints expression in clusters. (k) Nine immunotherapeutic drugs solely showing significant IC50 difference.

Referring to previous research, different clusters, known as subtypes, usually showed different immune microenvironments leading to different immunotherapeutic responses [21, 22]. With 16 necroptosis-related lncRNAs, we regrouped patients into two clusters by the ConsensusClusterPlus (CC) R package based on necroptosis-related lncRNAs expression (Figures 7(a) and S3A) [15]. T-distributed stochastic neighbor embedding (t-SNE) indicated two clusters could be distinguished clearly (Figure 7(b)). In addition, we employed principal component analyses (PCA) to verify that both risk groups and clusters have different PCA (Figure 7(c)). Moreover, cluster 1 had better OS (*p*=0.006) in the Kaplan–Meier analysis (Figure 7(d)). GSEA was also employed to investigate clusters' biological functions. Seven of the top ten pathways with enrichment in cluster 2 were related to immunity (*p* < 0.05; FDR < 0.25; |NES| > 1.9), such as “natural killer (NK) cell-mediated cytotoxicity” (Figures 7(e) and Figure S1B). NK cells and their cytotoxicity acted as an important role in immunity and cancer [23]. To verify its relations with risk, a chart was also made. Cluster 1 was significantly associated with the low-risk group, and cluster 2 was associated with the high-risk group (*∗∗* means *p* < 0.01) (Figure 7(f)). The results below about cluster 2 might contribute to patients' immunotherapy in risk groups. Concerning the comparison of the single sample GSEA (ssGSEA) scores for immune cells and immune functions, 15 immune cells, such as CD8^+^ T cells, and 12 immune functions, such as inflammation-promoting, had more relations with cluster 2 (Figure 7(g)). Cluster 2 was more highly infiltrated by immune cells based on analyses of the different platforms (Figure 7(h)) (Appendix D3). Cluster 2 had a higher immune score and a higher ESTIMAT (microenvironment) score, signifying a different TME from cluster 1 (Figure 7(i)). Almost all the immune checkpoints expressed more activity in cluster 2, such as HAVCR2 (TIM3), LAG3, and CD274 (PD-L1) (Figure 7(j)). CD8^+^ T cells, the function of inflammation-promoting, high immune score, activation of TIM3, LAG3, and PD-L1 played vital roles in the hot tumor. Therefore, we could consider cluster 2 as the hot tumor while cluster 1 as the cold tumor [5, 24]. It might result in different immunotherapeutic responses [22, 25]. With the notion of the cold and hot tumors, cluster 2 was more susceptible to immunotherapy. With drug sensitivity comparison, we found nine immunotherapeutic drugs, such as shikonin, showed different IC50 solely in clusters as well as 16 chemical or targeted drugs that applied to systemic treatments in GC (Figure 7(k) and Figure S3B) [26, 27]. Because of clusters based on these lncRNAs, we might further study immunotherapy responses and potentiate precise medication in GC patients.

## 4. Discussion

Immunotherapy can ameliorate the situation of frequently reported treatment failure, but it is not a panacea for all diseases [4]. Because of immunosuppressive TME, some patients had poor immunotherapy responses. Therefore, we introduced the notion that cold and hot tumors refer to an immune-based classification of tumors rather than conventional cancer-based for improving immunotherapy. The highly infiltrated tumor with a high immune score usually is regarded as the hot tumor while the noninfiltrated tumor with a low immune score as the cold tumor. The higher activity of checkpoints, such as TIM3 and LAG3, is also the one of characteristics of the hot tumor. In the face of the hot tumor, we can treat patients with T-cell-targeting immunotherapies, microbiome modulation, or other immunotherapeutic drugs. But it is not easy for the cold tumor because it fails to unleash preexisting immunity with low degree T cells. CD8 + T cells can kill cancer cells by releasing PRF1, GNLY, or GZM and break tolerance as a preexisting immune response, enhancing immunotherapy via the PD-1/PD-L1 immune inhibitory axis. So it is wise to turn a cold tumor into a hot tumor rather than just give other treatments [5, 28].

In the study, we built 16 necroptosis-related lncRNAs mode and tried to identify the cold and hot tumors. Patients were regrouped into low- and high-risk groups by the model and made some analyses such as Kaplan–Meier analysis, GSEA, and IC50 prediction. Although we found risk groups could be a guide in predicting prognoses and systemic treatments, we could not identify the hot tumor by risk groups. Referring to reports, molecular subtypes, also known as clusters, are associated with tumor immune suppression and microenvironments [1, 29]. Different subtypes have different immune and TME scores leading to different prognoses and immunotherapy responses [22, 25]. Thus, we divided patients into two clusters based on the expression of these lncRNAs [15]. As expected, the two clusters had different immune microenvironments. Cluster 1 had an immunosuppressive TME. At the same time, there were more CD8^+^ T cells highly infiltrated, more active function of inflammation-promoting, higher immune score, and higher activity of TIM3, LAG3, and PD-L1 in cluster 2, which could be identified as the hot tumor definitely [5, 24]. What's more, cluster 2 was more sensitive to immunotherapeutic drugs. Necroptosis-related lncRNAs could not only predict prognosis but also be a guide for individual therapy. Above all, these lncRNAs, as liquid biopsies, could distinguish between the cold and hot tumor briefly and effectively compared with the tumor biopsy by imaging mass cytometry or other experiments [28].

Besides, in the Sankey diagram, we found some of these lncRNAs were related to star genes such as BCL2. FRMD6-AS2 and LINC02657 were associated with AXL, which contributed to immunotherapy by reprogramming the immunological microenvironment and PD-1 inhibitors [30]. BCL2, as a hot point of PD-1 immunotherapy, correlated with AC012409.3 and AC069549.1. It also could reactive apoptosis to overcome immunotherapy failure and keep durable antitumor responses [31]. RNF31 was a potential immunotherapy target of GC and might be regulated by AC004596.1 [32]. TRAF2, associated with AL355574.1, could augment immunotherapy by increasing the susceptibility of tumors [33]. FRMD6-AS2 suppressed tumor growth, migration, and invasion via the Hippo signaling pathway [34]. And Hippo signaling pathway is engaged in programmed cell death by regulating kinds of target such as YAP [35]. LINC02657 was reported that its overexpression would keep cancer cells from programmed cell death by regulating SART3 [36]. The other lncRNAs were firstly discovered. Newly acquired necroptosis-related lncRNAs knowledge could help us develop a better mechanistic understanding of GC, which would bring a breakthrough into clinical practice.

There were still some shortcomings and deficiencies though we had utilized many methods to asset our model. As a retrospective study, it was susceptible to the inherent biases of this research paradigm [37]. Although the activation of checkpoints performed significantly between risk groups and clusters, we could not make a comparison of corresponding checkpoint inhibitors IC50, such as PD-1 inhibitors, as a result of insufficient data on GDSC. We had performed internal validation by the test and entire sets in the model, but it was difficult to do external validation for prognoses. Even if we had retrieved all information of GSE84437 series and GSE62254 series matrices from Gene Expression Omnibus (GEO) (https://www.ncbi.nlm.nih.gov/geo/), we could not get appropriate information of lncRNAs because commercial microarray data had biases and limitations compared with GTEx and TCGA. However, the immune cell bubble and the immune cell heat map showed the results from multiple platforms, which might be recognized as external validation in a sense. Given the above analyses and previous reports, we felt that our model was reasonable and acceptable for future clinical tests [13, 38]. Collecting more clinical datasets could help reassert the value of these necroptosis-related lncRNAs, which would be in our plans.

In addition, both necroptosis and lncRNAs work in inducing cell death. Necroptosis can induce cancer cell death, bypassing apoptosis because of modality in a caspase-independent fashion [7]. LncRNAs can regulate apoptosis-related signaling pathways [39]. Clarifying their relations and mechanisms through experiments will be helpful for effectively killing cancer cells while leaving the healthy cells intact [7, 10]. It will make progress not only in immunotherapy but also in cancer research.

## 5. Conclusions

Necroptosis-related lncRNAs could predict prognosis and help propose an exploitable therapeutic strategy by identifying the cold and hot tumors, which would make great progress in individual therapy and improve patients' prognoses. Targeting necroptosis and lncRNAs will be a promising pathway for overcoming systemic treatments failure and expanding the field of immunotherapy. Therefore, the mechanisms and relationships, among necroptosis, lncRNAs, immunity, and GC, were worth being fully elucidated and validated.

## Data Availability

The data used to support the results are available at the TCGA (https://tcga-data.nci.nih.gov/tcga/), GTEx (https://www.gtexportal.org/), GSEA (http://www.gsea-msigdb.org/gsea/index.jsp), and GDSC (https://www.cancerrxgene.org/).

## Supplementary Materials

Supplementary Materials Appendix S1. Figure S1: the GSEA of the high-risk group and cluster 2. (A) The GSEA of the high-risk group. (B) The GSEA of cluster 2. Appendix S2. Figure S2: the IC50 prediction of 16 chemical or targeted drugs in risk groups. Appendix S3. Figure S3: consensus clustering analysis of necroptosis-related lncRNAs and IC50 prediction in clusters. (A) The heat map, cumulative distribution function (CDF) plot, and the consensus CDF plots of consensus clustering matrix. (B) 16 chemical or targeted drugs solely showing significant IC50 difference in clusters. Appendix T1: the table of necroptosis-related genes refers to GSEA and previous reports. Appendix D1: the network data of necroptosis-related genes and lncRNAs. Appendix D2: the profile of significantly differently infiltrated immune cells between risk groups in different platforms. Appendix D3: the profile of significantly differently infiltrated immune cells between clusters in different platforms.
