# Supplementary material for: Necroptosis-Related lncRNAs: Predicting Prognosis and the Distinction between the Cold and Hot Tumors in Gastric Cancer
Source: J Oncol. 2021 Nov 8;2021:6718443. doi: 10.1155/2021/6718443 (PMC8592775; doi:10.1155/2021/6718443)
Supplement: Supplementary Materials — Appendix S1. Figure S1: the GSEA of the high-risk group and cluster 2. (A) The GSEA of the high-risk group. (B) The GSEA of cluster 2. Appendix S2. Figure S2: the IC50 prediction of 16 chemical or targeted drugs in risk groups. Appendix S3. Figure S3: consensus clustering analysis of necroptosis-related lncRNAs and IC50 prediction in clusters. (A) The heat map, cumulative distribution function (CDF) plot, and the consensus CDF plots of consensus clustering matrix. (B) 16 chemical or targeted drugs solely showing significant IC50 difference in clusters. Appendix T1: the table of necroptosis-related genes refers to GSEA and previous reports. Appendix D1: the network data of necroptosis-related genes and lncRNAs. Appendix D2: the profile of significantly differently infiltrated immune cells between risk groups in different platforms. Appendix D3: the profile of significantly differently infiltrated immune cells between clusters in different platforms. [file 6718443.f1.zip › 6718443.f1/Appendix D1.docx]

| Necroptosis gene | lncRNA | cor | pvalue | Regulation |
| --- | --- | --- | --- | --- |
| FASLG | AC116914.1 | 0.603505235 | 2.07E-35 | postive |
| ZBP1 | AC116914.1 | 0.467937275 | 4.58E-20 | postive |
| DIABLO | AC067817.2 | -0.409621739 | 2.61E-15 | negative |
| KLF9 | AC005165.1 | 0.504243988 | 1.60E-23 | postive |
| TRIM11 | MIR100HG | -0.491818486 | 2.72E-22 | negative |
| AXL | MIR100HG | 0.559804308 | 1.11E-29 | postive |
| BCL2 | MIR100HG | 0.491932442 | 2.65E-22 | postive |
| PLK1 | MIR100HG | -0.461606755 | 1.67E-19 | negative |
| KLF9 | MIR100HG | 0.698405971 | 1.79E-51 | postive |
| IDH2 | AC018946.1 | 0.423071174 | 2.51E-16 | postive |
| DDX58 | AP001610.2 | 0.518543479 | 5.33E-25 | postive |
| CYLD | AC092803.1 | -0.402804255 | 8.22E-15 | negative |
| KLF9 | AC139768.1 | 0.40866582 | 3.07E-15 | postive |
| FASLG | LINC02195 | 0.692010969 | 3.40E-50 | postive |
| ZBP1 | LINC02195 | 0.424766942 | 1.85E-16 | postive |
| TNFSF10 | LINC02195 | 0.47986773 | 3.72E-21 | postive |
| AXL | AP000941.1 | 0.449629155 | 1.79E-18 | postive |
| KLF9 | AP000941.1 | 0.480249985 | 3.43E-21 | postive |
| CYLD | AL157402.2 | 0.426688697 | 1.31E-16 | postive |
| BCL2 | AL157402.2 | 0.450604588 | 1.48E-18 | postive |
| FLT3 | AL157402.2 | 0.403037858 | 7.90E-15 | postive |
| BACH2 | AL157402.2 | 0.49671751 | 9.03E-23 | postive |
| ATRX | AL353804.2 | 0.442844517 | 6.60E-18 | postive |
| FAS | AC004847.1 | 0.404676191 | 6.01E-15 | postive |
| HDAC9 | AC004847.1 | 0.627367707 | 6.27E-39 | postive |
| ZBP1 | AC133644.1 | 0.459057997 | 2.79E-19 | postive |
| BCL2 | PGM5P4-AS1 | 0.467349327 | 5.17E-20 | postive |
| KLF9 | PGM5P4-AS1 | 0.466671419 | 5.95E-20 | postive |
| AXL | LINC01615 | 0.43119726 | 5.79E-17 | postive |
| ATRX | TRMT2B-AS1 | 0.402555306 | 8.56E-15 | postive |
| TRIM11 | ADAMTS9-AS2 | -0.409854802 | 2.51E-15 | negative |
| AXL | ADAMTS9-AS2 | 0.523999647 | 1.39E-25 | postive |
| BCL2 | ADAMTS9-AS2 | 0.511022438 | 3.26E-24 | postive |
| PLK1 | ADAMTS9-AS2 | -0.434614689 | 3.09E-17 | negative |
| KLF9 | ADAMTS9-AS2 | 0.82291955 | 9.85E-86 | postive |
| KLF9 | AC055874.1 | 0.487929628 | 6.44E-22 | postive |
| RNF31 | AC004596.1 | 0.409151221 | 2.83E-15 | postive |
| FAS | USP30-AS1 | 0.425162802 | 1.73E-16 | postive |
| FASLG | USP30-AS1 | 0.712384692 | 2.18E-54 | postive |
| TLR3 | USP30-AS1 | 0.429945908 | 7.28E-17 | postive |
| ZBP1 | USP30-AS1 | 0.547652935 | 3.10E-28 | postive |
| TNFSF10 | USP30-AS1 | 0.458380449 | 3.19E-19 | postive |
| CD40 | USP30-AS1 | 0.486674318 | 8.49E-22 | postive |
| TRAF2 | AL355574.1 | 0.425090051 | 1.75E-16 | postive |
| KLF9 | AC121247.2 | 0.42879697 | 8.97E-17 | postive |
| CYLD | TRPM2-AS | -0.448486602 | 2.24E-18 | negative |
| FASLG | AL445471.1 | 0.409949446 | 2.47E-15 | postive |
| CYLD | AP005131.4 | 0.508496871 | 5.92E-24 | postive |
| CFLAR | AP005131.4 | 0.445016824 | 4.36E-18 | postive |
| BCL2 | AP005131.4 | 0.658409707 | 5.44E-44 | postive |
| FLT3 | AP005131.4 | 0.491341591 | 3.02E-22 | postive |
| BACH2 | AP005131.4 | 0.548569646 | 2.42E-28 | postive |
| KLF9 | AP005131.4 | 0.404369799 | 6.33E-15 | postive |
| TARDBP | AC018809.1 | 0.482658361 | 2.04E-21 | postive |
| KLF9 | AC027807.2 | 0.493504468 | 1.86E-22 | postive |
| TARDBP | AL109811.2 | 0.544520861 | 7.15E-28 | postive |
| AXL | AC090044.1 | 0.428400924 | 9.64E-17 | postive |
| BCL2 | AC090044.1 | 0.471939618 | 1.99E-20 | postive |
| KLF9 | AC090044.1 | 0.741586633 | 4.41E-61 | postive |
| AXL | MIR1-1HG-AS1 | 0.474464136 | 1.17E-20 | postive |
| BCL2 | MIR1-1HG-AS1 | 0.457446744 | 3.85E-19 | postive |
| KLF9 | MIR1-1HG-AS1 | 0.783088536 | 2.52E-72 | postive |
| AXL | LINC01711 | 0.620546724 | 6.83E-38 | postive |
| AXL | GAS1RR | 0.454159416 | 7.39E-19 | postive |
| BCL2 | GAS1RR | 0.460716349 | 2.00E-19 | postive |
| KLF9 | GAS1RR | 0.681087181 | 4.36E-48 | postive |
| DIABLO | AL049835.1 | -0.419391308 | 4.81E-16 | negative |
| AXL | LINC02613 | 0.415533014 | 9.45E-16 | postive |
| BCL2 | LINC02613 | 0.467221113 | 5.31E-20 | postive |
| KLF9 | LINC02613 | 0.722562252 | 1.27E-56 | postive |
| CYLD | MAFG-DT | -0.401516662 | 1.02E-14 | negative |
| CYLD | AC005674.2 | 0.421719029 | 3.19E-16 | postive |
| KLF9 | NALT1 | 0.681018631 | 4.50E-48 | postive |
| KLF9 | LINC01783 | 0.430729199 | 6.31E-17 | postive |
| DIABLO | AC090559.2 | -0.422871533 | 2.60E-16 | negative |
| ATRX | AC090559.2 | 0.412667256 | 1.55E-15 | postive |
| ATRX | AC025043.1 | 0.450667998 | 1.46E-18 | postive |
| TARDBP | RNF139-AS1 | 0.449752758 | 1.75E-18 | postive |
| TSC1 | AL513327.1 | 0.405735341 | 5.03E-15 | postive |
| AXL | AC009102.2 | 0.451702426 | 1.20E-18 | postive |
| BCL2 | AC009102.2 | 0.471828111 | 2.04E-20 | postive |
| KLF9 | AC009102.2 | 0.733726231 | 3.41E-59 | postive |
| DIABLO | AC004000.1 | -0.470231574 | 2.85E-20 | negative |
| ATRX | AC004000.1 | 0.44170633 | 8.20E-18 | postive |
| HDAC9 | AL391069.2 | 0.423724721 | 2.23E-16 | postive |
| TARDBP | AC007390.1 | 0.448595158 | 2.19E-18 | postive |
| AXL | AC025165.1 | 0.50361156 | 1.86E-23 | postive |
| BCL2 | AC025165.1 | 0.456794796 | 4.38E-19 | postive |
| KLF9 | AC025165.1 | 0.771043218 | 8.10E-69 | postive |
| TRIM11 | AC012409.3 | -0.405379544 | 5.34E-15 | negative |
| BCL2 | AC012409.3 | 0.444276919 | 5.03E-18 | postive |
| KLF9 | AC012409.3 | 0.594913357 | 3.25E-34 | postive |
| AXL | MIR497HG | 0.426495668 | 1.36E-16 | postive |
| BCL2 | MIR497HG | 0.470943387 | 2.46E-20 | postive |
| KLF9 | MIR497HG | 0.630348717 | 2.17E-39 | postive |
| CYLD | AC087500.1 | 0.401257216 | 1.06E-14 | postive |
| CFLAR | AC087500.1 | 0.430439551 | 6.65E-17 | postive |
| BCL2 | AC087500.1 | 0.603043905 | 2.41E-35 | postive |
| FLT3 | AC087500.1 | 0.411876966 | 1.77E-15 | postive |
| BACH2 | AC087500.1 | 0.484486531 | 1.37E-21 | postive |
| ATRX | AC087500.1 | 0.479077568 | 4.41E-21 | postive |
| KLF9 | AC087500.1 | 0.403671607 | 7.11E-15 | postive |
| ID1 | BX470102.1 | 0.454545737 | 6.84E-19 | postive |
| DIABLO | AC023389.2 | -0.418436738 | 5.69E-16 | negative |
| ATRX | AC023389.2 | 0.430645073 | 6.41E-17 | postive |
| DIABLO | SND1-IT1 | -0.462503674 | 1.39E-19 | negative |
| ATRX | AC020915.2 | 0.441742421 | 8.14E-18 | postive |
| CYLD | AC107959.1 | 0.415229548 | 9.96E-16 | postive |
| AXL | AC107959.1 | 0.434977478 | 2.89E-17 | postive |
| BCL2 | AC107959.1 | 0.52101184 | 2.91E-25 | postive |
| FLT3 | AC107959.1 | 0.41751194 | 6.69E-16 | postive |
| KLF9 | AC107959.1 | 0.618982927 | 1.17E-37 | postive |
| KLF9 | OXCT1-AS1 | 0.466724452 | 5.88E-20 | postive |
| RNF31 | AC073569.2 | 0.401802777 | 9.70E-15 | postive |
| KLF9 | AC036108.3 | 0.58805597 | 2.76E-33 | postive |
| TSC1 | AL157392.3 | 0.457697709 | 3.66E-19 | postive |
| AXL | AL139220.2 | 0.454097401 | 7.48E-19 | postive |
| KLF9 | AL139220.2 | 0.53828233 | 3.69E-27 | postive |
| BCL2 | AP005131.2 | 0.421236381 | 3.48E-16 | postive |
| DIABLO | AC134407.1 | -0.447075416 | 2.94E-18 | negative |
| ATRX | AC134407.1 | 0.519089454 | 4.66E-25 | postive |
| KLF9 | COL4A2-AS1 | 0.547047408 | 3.64E-28 | postive |
| ALK | AL009178.2 | 0.465265963 | 7.93E-20 | postive |
| KLF9 | AP000894.2 | 0.574505538 | 1.63E-31 | postive |
| CYLD | SH3BP5-AS1 | 0.452481804 | 1.03E-18 | postive |
| CFLAR | SH3BP5-AS1 | 0.438479089 | 1.51E-17 | postive |
| ATRX | SH3BP5-AS1 | 0.455195952 | 6.02E-19 | postive |
| GATA3 | LINC01322 | 0.446509552 | 3.28E-18 | postive |
| CFLAR | AC009120.2 | 0.411165393 | 2.00E-15 | postive |
| ATRX | AC009120.2 | 0.491003759 | 3.26E-22 | postive |
| OTULIN | AC114956.2 | 0.440022279 | 1.13E-17 | postive |
| TARDBP | AP002449.1 | 0.413680289 | 1.30E-15 | postive |
| CYLD | AL513008.1 | 0.433046622 | 4.13E-17 | postive |
| CFLAR | AL513008.1 | 0.424480517 | 1.95E-16 | postive |
| BCL2 | AL513008.1 | 0.559324812 | 1.27E-29 | postive |
| FLT3 | AL513008.1 | 0.451262165 | 1.30E-18 | postive |
| BACH2 | AL513008.1 | 0.641009032 | 4.41E-41 | postive |
| ATRX | AC087501.1 | 0.455852236 | 5.28E-19 | postive |
| DIABLO | REV3L-IT1 | -0.406874801 | 4.16E-15 | negative |
| DNMT1 | AL137058.2 | 0.422637786 | 2.71E-16 | postive |
| GATA3 | UBXN10-AS1 | 0.402097823 | 9.24E-15 | postive |
| KLF9 | UBXN10-AS1 | 0.577001733 | 7.81E-32 | postive |
| BCL2 | AL356599.1 | 0.464214426 | 9.83E-20 | postive |
| KLF9 | AL356599.1 | 0.660058779 | 2.81E-44 | postive |
| CYLD | AL139289.1 | -0.411275932 | 1.97E-15 | negative |
| AXL | AL513217.1 | 0.444055455 | 5.24E-18 | postive |
| BCL2 | AL513217.1 | 0.419225524 | 4.96E-16 | postive |
| KLF9 | AL513217.1 | 0.748329694 | 9.32E-63 | postive |
| CYLD | AL031846.2 | 0.568549889 | 9.25E-31 | postive |
| CFLAR | AL031846.2 | 0.502841674 | 2.22E-23 | postive |
| BCL2 | AL031846.2 | 0.648072015 | 3.07E-42 | postive |
| FLT3 | AL031846.2 | 0.466421333 | 6.26E-20 | postive |
| PLK1 | AL031846.2 | -0.407210192 | 3.93E-15 | negative |
| BACH2 | AL031846.2 | 0.422168028 | 2.95E-16 | postive |
| KLF9 | AL031846.2 | 0.560224846 | 9.85E-30 | postive |
| DIABLO | AC080162.1 | -0.405982704 | 4.83E-15 | negative |
| ATRX | AC080162.1 | 0.493150245 | 2.02E-22 | postive |
| AXL | AJ011932.1 | 0.464991864 | 8.39E-20 | postive |
| KLF9 | AJ011932.1 | 0.561936261 | 6.09E-30 | postive |
| DIABLO | AC005072.1 | -0.410301159 | 2.32E-15 | negative |
| AXL | AC245041.1 | 0.573671073 | 2.09E-31 | postive |
| AXL | AL355512.1 | 0.499448277 | 4.85E-23 | postive |
| DIABLO | AC006441.1 | -0.454499309 | 6.91E-19 | negative |
| ATRX | AC006441.1 | 0.403674547 | 7.11E-15 | postive |
| ATRX | AC084824.4 | 0.448507523 | 2.23E-18 | postive |
| CYLD | AC138696.2 | -0.436779608 | 2.07E-17 | negative |
| DNMT1 | AC008752.3 | 0.588072742 | 2.75E-33 | postive |
| KLF9 | AC112503.2 | 0.436176883 | 2.31E-17 | postive |
| FASLG | TRBV11-2 | 0.459126993 | 2.75E-19 | postive |
| ZBP1 | TRBV11-2 | 0.410858769 | 2.11E-15 | postive |
| TNFRSF1B | TRBV11-2 | 0.459354353 | 2.63E-19 | postive |
| CYLD | TRBV11-2 | 0.451552661 | 1.23E-18 | postive |
| BCL2 | TRBV11-2 | 0.435377419 | 2.68E-17 | postive |
| FLT3 | TRBV11-2 | 0.627214278 | 6.62E-39 | postive |
| BACH2 | TRBV11-2 | 0.466417282 | 6.26E-20 | postive |
| CD40 | TRBV11-2 | 0.435068631 | 2.84E-17 | postive |
| ATRX | RNF216-IT1 | 0.512112642 | 2.51E-24 | postive |
| KLF9 | RNF216-IT1 | 0.478387446 | 5.11E-21 | postive |
| BACH2 | AC025279.1 | 0.411810814 | 1.80E-15 | postive |
| HDAC9 | AC025279.1 | 0.563231515 | 4.22E-30 | postive |
| CYLD | AC011816.2 | 0.465870685 | 7.01E-20 | postive |
| BCL2 | AC011816.2 | 0.419886578 | 4.41E-16 | postive |
| FLT3 | AC011816.2 | 0.410020682 | 2.44E-15 | postive |
| BACH2 | AC011816.2 | 0.485077675 | 1.20E-21 | postive |
| AXL | AC009549.1 | 0.61539605 | 3.99E-37 | postive |
| KLF9 | AC009549.1 | 0.534052725 | 1.10E-26 | postive |
| ATRX | AP002812.3 | 0.40486073 | 5.83E-15 | postive |
| KLF9 | ZNF667-AS1 | 0.520248602 | 3.51E-25 | postive |
| DNMT1 | MRPL23-AS1 | 0.575939165 | 1.07E-31 | postive |
| CDKN2A | AL606970.1 | 0.418870257 | 5.27E-16 | postive |
| CYLD | AC010149.1 | 0.527185775 | 6.29E-26 | postive |
| CFLAR | AC010149.1 | 0.497160779 | 8.16E-23 | postive |
| BCL2 | AC010149.1 | 0.432688015 | 4.41E-17 | postive |
| FLT3 | AC010149.1 | 0.522625894 | 1.96E-25 | postive |
| BACH2 | AC010149.1 | 0.497559879 | 7.46E-23 | postive |
| TARDBP | PTOV1-AS2 | 0.438606868 | 1.47E-17 | postive |
| TARDBP | ZBTB40-IT1 | 0.415530173 | 9.45E-16 | postive |
| ATRX | AL450344.2 | 0.445045016 | 4.34E-18 | postive |
| ATRX | AC025430.1 | 0.411903639 | 1.77E-15 | postive |
| DNMT1 | AL161891.1 | 0.550451799 | 1.46E-28 | postive |
| AXL | LINC01537 | 0.417543811 | 6.65E-16 | postive |
| BCL2 | LINC01537 | 0.404472026 | 6.22E-15 | postive |
| KLF9 | LINC01537 | 0.682998024 | 1.90E-48 | postive |
| CYLD | AC010609.1 | 0.511911485 | 2.64E-24 | postive |
| CFLAR | AC010609.1 | 0.449238264 | 1.93E-18 | postive |
| BCL2 | AC010609.1 | 0.551295407 | 1.16E-28 | postive |
| FLT3 | AC010609.1 | 0.467451764 | 5.06E-20 | postive |
| BACH2 | AC010609.1 | 0.582315525 | 1.59E-32 | postive |
| TRAF2 | AL161452.1 | 0.448885144 | 2.07E-18 | postive |
| CYLD | AC079921.1 | 0.542639012 | 1.18E-27 | postive |
| STAT3 | AC079921.1 | 0.408165411 | 3.34E-15 | postive |
| CFLAR | AC079921.1 | 0.419202017 | 4.98E-16 | postive |
| BCL2 | AC079921.1 | 0.44434362 | 4.96E-18 | postive |
| DIABLO | AL049840.6 | -0.429615939 | 7.73E-17 | negative |
| FASLG | AP002954.1 | 0.446917145 | 3.03E-18 | postive |
| HDAC9 | AC136424.2 | 0.530927835 | 2.44E-26 | postive |
| ATRX | AC018410.1 | 0.401424589 | 1.03E-14 | postive |
| ATRX | AC005154.4 | 0.436096453 | 2.35E-17 | postive |
| CYLD | AC020913.1 | 0.424469877 | 1.96E-16 | postive |
| CFLAR | AC020913.1 | 0.419286957 | 4.90E-16 | postive |
| DIABLO | AL590093.1 | -0.41372269 | 1.29E-15 | negative |
| ATRX | AL590093.1 | 0.412352781 | 1.64E-15 | postive |
| DIABLO | AL158163.1 | -0.422768696 | 2.65E-16 | negative |
| ATRX | AL158163.1 | 0.510445529 | 3.73E-24 | postive |
| AXL | LINC02256 | 0.41734037 | 6.89E-16 | postive |
| BCL2 | LINC02256 | 0.519571612 | 4.14E-25 | postive |
| PLK1 | LINC02256 | -0.434609886 | 3.09E-17 | negative |
| KLF9 | LINC02256 | 0.633931138 | 5.96E-40 | postive |
| TRIM11 | AC093010.2 | -0.477722142 | 5.88E-21 | negative |
| DIABLO | AC093010.2 | -0.457126383 | 4.10E-19 | negative |
| CFLAR | AC093010.2 | 0.462003889 | 1.54E-19 | postive |
| AXL | AC093010.2 | 0.467690779 | 4.82E-20 | postive |
| BCL2 | AC093010.2 | 0.528702348 | 4.29E-26 | postive |
| PLK1 | AC093010.2 | -0.585277406 | 6.47E-33 | negative |
| ATRX | AC093010.2 | 0.571559274 | 3.87E-31 | postive |
| KLF9 | AC093010.2 | 0.627967445 | 5.07E-39 | postive |
| KLF9 | LINC01140 | 0.406828263 | 4.19E-15 | postive |
| ATRX | AC120349.1 | 0.456383876 | 4.75E-19 | postive |
| CFLAR | AC120193.1 | 0.422986861 | 2.55E-16 | postive |
| BCL2 | AC120193.1 | 0.40815204 | 3.35E-15 | postive |
| FLT3 | AC120193.1 | 0.445715262 | 3.82E-18 | postive |
| CYLD | AP004609.3 | 0.466488623 | 6.17E-20 | postive |
| BCL2 | AP004609.3 | 0.60573172 | 1.00E-35 | postive |
| BACH2 | AP004609.3 | 0.402034045 | 9.34E-15 | postive |
| KLF9 | AP004609.3 | 0.554814828 | 4.42E-29 | postive |
| DIABLO | AC002558.3 | -0.480148259 | 3.50E-21 | negative |
| ATRX | AC002558.3 | 0.506848008 | 8.73E-24 | postive |
| SPATA2 | AL078587.1 | 0.42028185 | 4.11E-16 | postive |
| DIABLO | AC245060.5 | -0.422555684 | 2.75E-16 | negative |
| ATRX | AC245060.5 | 0.450163709 | 1.62E-18 | postive |
| ATRX | AC130650.1 | 0.41721755 | 7.04E-16 | postive |
| KLF9 | AC130650.1 | 0.439091524 | 1.34E-17 | postive |
| BCL2 | AL021368.2 | 0.447722811 | 2.59E-18 | postive |
| AXL | LNCAROD | 0.438340484 | 1.54E-17 | postive |
| ID1 | AC068580.2 | 0.411872742 | 1.78E-15 | postive |
| DIABLO | HM13-IT1 | -0.405173574 | 5.53E-15 | negative |
| AXL | AC002398.2 | 0.468240583 | 4.30E-20 | postive |
| BCL2 | AC002398.2 | 0.451549708 | 1.23E-18 | postive |
| KLF9 | AC002398.2 | 0.687626004 | 2.45E-49 | postive |
| BACH2 | AC023510.1 | 0.501529124 | 3.01E-23 | postive |
| TERT | AC129492.1 | 0.464718355 | 8.87E-20 | postive |
| AXL | CYP1B1-AS1 | 0.451034862 | 1.36E-18 | postive |
| BCL2 | CYP1B1-AS1 | 0.533875765 | 1.15E-26 | postive |
| KLF9 | CYP1B1-AS1 | 0.652194931 | 6.26E-43 | postive |
| RNF31 | AC073046.1 | 0.416676424 | 7.74E-16 | postive |
| TSC1 | AL162586.1 | 0.428145665 | 1.01E-16 | postive |
| RNF31 | AC073896.4 | -0.435407734 | 2.67E-17 | negative |
| STUB1 | THUMPD3-AS1 | -0.464047575 | 1.02E-19 | negative |
| MPG | THUMPD3-AS1 | -0.402311959 | 8.92E-15 | negative |
| TARDBP | THUMPD3-AS1 | 0.513121881 | 1.97E-24 | postive |
| KLF9 | AC103770.1 | 0.458542708 | 3.09E-19 | postive |
| FASLG | AC011511.5 | 0.470545398 | 2.67E-20 | postive |
| TERT | AC011511.5 | 0.424152094 | 2.07E-16 | postive |
| TARDBP | AP000442.1 | 0.506832088 | 8.76E-24 | postive |
| KLF9 | LINC01266 | 0.437366685 | 1.85E-17 | postive |
| BNIP3 | LINC01266 | 0.406445613 | 4.47E-15 | postive |
| ATRX | AL590006.1 | 0.411265341 | 1.97E-15 | postive |
| LEF1 | FZD10-AS1 | 0.416295298 | 8.27E-16 | postive |
| KLF9 | AC112250.2 | 0.600874152 | 4.86E-35 | postive |
| CFLAR | AC136604.2 | 0.428491676 | 9.48E-17 | postive |
| BCL2 | AC136604.2 | 0.412619488 | 1.56E-15 | postive |
| ATRX | AC136604.2 | 0.464024558 | 1.02E-19 | postive |
| KLF9 | AC136604.2 | 0.544229376 | 7.73E-28 | postive |
| KLF9 | FAM66C | 0.440099984 | 1.11E-17 | postive |
| DIABLO | AP005131.5 | -0.429220059 | 8.31E-17 | negative |
| BCL2 | AP005131.5 | 0.402281133 | 8.96E-15 | postive |
| ATRX | AP005131.5 | 0.40802521 | 3.42E-15 | postive |
| KLF9 | AC110491.1 | 0.526638398 | 7.21E-26 | postive |
| BCL2 | LINC02185 | 0.423786876 | 2.21E-16 | postive |
| KLF9 | LINC02185 | 0.589567062 | 1.73E-33 | postive |
| CYLD | AC010226.1 | 0.405800224 | 4.98E-15 | postive |
| BCL2 | AC010226.1 | 0.4632067 | 1.21E-19 | postive |
| TARDBP | AC130650.2 | 0.403850623 | 6.90E-15 | postive |
| FADD | AP000879.2 | 0.455021551 | 6.23E-19 | postive |
| DIABLO | AC010834.3 | -0.410444398 | 2.27E-15 | negative |
| FASLG | AC090912.2 | 0.458536448 | 3.10E-19 | postive |
| HDAC9 | LINC02542 | 0.618071842 | 1.60E-37 | postive |
| AXL | AC080038.2 | 0.530439867 | 2.77E-26 | postive |
| KLF9 | AC080038.2 | 0.439039866 | 1.35E-17 | postive |
| CYLD | PSMD6-AS2 | 0.451212145 | 1.32E-18 | postive |
| FASLG | AC007278.1 | 0.408857839 | 2.97E-15 | postive |
| TNFRSF1B | AC007278.1 | 0.414803332 | 1.07E-15 | postive |
| KLF9 | AC103740.1 | 0.529569721 | 3.45E-26 | postive |
| AXL | AC026355.2 | 0.42315034 | 2.47E-16 | postive |
| TRIM11 | ACTA2-AS1 | -0.450884664 | 1.40E-18 | negative |
| AXL | ACTA2-AS1 | 0.495304123 | 1.24E-22 | postive |
| BCL2 | ACTA2-AS1 | 0.463005132 | 1.26E-19 | postive |
| PLK1 | ACTA2-AS1 | -0.408297675 | 3.27E-15 | negative |
| KLF9 | ACTA2-AS1 | 0.678514198 | 1.33E-47 | postive |
| AXL | NR2F2-AS1 | 0.457659635 | 3.69E-19 | postive |
| KLF9 | NR2F2-AS1 | 0.553853345 | 5.76E-29 | postive |
| CYLD | AL133371.2 | 0.582675501 | 1.43E-32 | postive |
| BCL2 | AL133371.2 | 0.617772492 | 1.77E-37 | postive |
| FLT3 | AL133371.2 | 0.678725582 | 1.21E-47 | postive |
| BACH2 | AL133371.2 | 0.639295369 | 8.34E-41 | postive |
| AXL | AC135012.3 | 0.428215985 | 9.97E-17 | postive |
| BCL2 | AC135012.3 | 0.487578627 | 6.96E-22 | postive |
| FLT3 | AC135012.3 | 0.411492949 | 1.90E-15 | postive |
| KLF9 | AC135012.3 | 0.623890821 | 2.13E-38 | postive |
| TARDBP | SNHG12 | 0.452354745 | 1.05E-18 | postive |
| TARDBP | AC007038.1 | 0.515493124 | 1.12E-24 | postive |
| ATRX | AC090519.2 | 0.441754513 | 8.12E-18 | postive |
| AXL | MYO16-AS1 | 0.456986819 | 4.22E-19 | postive |
| TSC1 | AL132989.1 | 0.439391332 | 1.27E-17 | postive |
| CYLD | AL132989.1 | 0.402330682 | 8.89E-15 | postive |
| CFLAR | AL132989.1 | 0.440741284 | 9.84E-18 | postive |
| ATRX | AL132989.1 | 0.48492242 | 1.25E-21 | postive |
| ATRX | AC055855.2 | 0.408429013 | 3.20E-15 | postive |
| BCL2 | AC018752.1 | 0.421350166 | 3.41E-16 | postive |
| KLF9 | AC018752.1 | 0.538489676 | 3.50E-27 | postive |
| DIABLO | AL138963.1 | -0.418859476 | 5.28E-16 | negative |
| ATRX | AL683813.2 | 0.402198445 | 9.09E-15 | postive |
| SQSTM1 | AC004148.1 | -0.412617002 | 1.56E-15 | negative |
| STUB1 | AC004148.1 | -0.462211531 | 1.48E-19 | negative |
| TARDBP | AC004148.1 | 0.531236678 | 2.26E-26 | postive |
| PLK1 | AC004943.2 | 0.416729816 | 7.67E-16 | postive |
| FASLG | AC083862.1 | 0.522791163 | 1.88E-25 | postive |
| ZBP1 | AC083862.1 | 0.495583737 | 1.17E-22 | postive |
| TNFSF10 | AC083862.1 | 0.497300986 | 7.91E-23 | postive |
| CYLD | AC083862.1 | 0.406949497 | 4.10E-15 | postive |
| FAS | MIR155HG | 0.428213653 | 9.97E-17 | postive |
| FASLG | MIR155HG | 0.417836616 | 6.32E-16 | postive |
| ZBP1 | MIR155HG | 0.482579122 | 2.07E-21 | postive |
| CYLD | MIR155HG | 0.437411231 | 1.84E-17 | postive |
| FLT3 | MIR155HG | 0.426761539 | 1.30E-16 | postive |
| BACH2 | MIR155HG | 0.51290048 | 2.08E-24 | postive |
| BNIP3 | AL592424.1 | 0.416453166 | 8.05E-16 | postive |
| CYLD | ELF3-AS1 | -0.464598915 | 9.09E-20 | negative |
| BCL2 | ELF3-AS1 | -0.431084551 | 5.91E-17 | negative |
| CYLD | AC025917.1 | 0.580596788 | 2.67E-32 | postive |
| CFLAR | AC025917.1 | 0.458729472 | 2.98E-19 | postive |
| BCL2 | AC025917.1 | 0.410759648 | 2.15E-15 | postive |
| BACH2 | AC025917.1 | 0.445941124 | 3.66E-18 | postive |
| ATRX | AC025917.1 | 0.422269017 | 2.89E-16 | postive |
| FADD | AL096828.1 | 0.484458042 | 1.38E-21 | postive |
| DNMT1 | AL096828.1 | 0.721284817 | 2.45E-56 | postive |
| KLF9 | AL356489.2 | 0.580977404 | 2.38E-32 | postive |
| AXL | HAND2-AS1 | 0.476701473 | 7.31E-21 | postive |
| BCL2 | HAND2-AS1 | 0.445713448 | 3.82E-18 | postive |
| KLF9 | HAND2-AS1 | 0.758896352 | 1.71E-65 | postive |
| TLR3 | AC098848.1 | 0.406998334 | 4.07E-15 | postive |
| DIABLO | AP005131.3 | -0.440723758 | 9.87E-18 | negative |
| BCL2 | AP005131.3 | 0.452183922 | 1.09E-18 | postive |
| BACH2 | AP005131.3 | 0.406322075 | 4.56E-15 | postive |
| ATRX | AP005131.3 | 0.416716462 | 7.69E-16 | postive |
| DIABLO | AC024267.4 | -0.507959321 | 6.72E-24 | negative |
| ATRX | AC024267.4 | 0.498508123 | 6.01E-23 | postive |
| MYCN | AC003991.2 | 0.426868197 | 1.27E-16 | postive |
| CYLD | AC007496.1 | 0.421856588 | 3.11E-16 | postive |
| ATRX | AC007496.1 | 0.441150731 | 9.10E-18 | postive |
| DIABLO | AL139120.1 | -0.433149834 | 4.05E-17 | negative |
| ATRX | AL139120.1 | 0.458795144 | 2.94E-19 | postive |
| KLF9 | LINC02269 | 0.458024799 | 3.43E-19 | postive |
| TARDBP | AC108727.1 | 0.432390043 | 4.66E-17 | postive |
| TRIM11 | AL049838.1 | -0.400070209 | 1.29E-14 | negative |
| KLF9 | AL049838.1 | 0.501355601 | 3.13E-23 | postive |
| MYCN | DPH6-DT | 0.674805196 | 6.47E-47 | postive |
| BNIP3 | LINC01018 | 0.41818804 | 5.94E-16 | postive |
| KLF9 | ADAMTS9-AS1 | 0.644405802 | 1.23E-41 | postive |
| FADD | AL139339.1 | 0.438023122 | 1.64E-17 | postive |
| DNMT1 | AL139339.1 | 0.631145499 | 1.63E-39 | postive |
| CYLD | AC114760.2 | 0.527600583 | 5.66E-26 | postive |
| CFLAR | AC114760.2 | 0.478812971 | 4.66E-21 | postive |
| BCL2 | AC114760.2 | 0.611195894 | 1.64E-36 | postive |
| FLT3 | AC114760.2 | 0.492740669 | 2.21E-22 | postive |
| BACH2 | AC114760.2 | 0.701914448 | 3.45E-52 | postive |
| TRIM11 | FENDRR | -0.427258417 | 1.18E-16 | negative |
| AXL | FENDRR | 0.50171227 | 2.88E-23 | postive |
| BCL2 | FENDRR | 0.472990612 | 1.60E-20 | postive |
| PLK1 | FENDRR | -0.415105732 | 1.02E-15 | negative |
| KLF9 | FENDRR | 0.786598382 | 2.18E-73 | postive |
| OTULIN | AC010491.1 | 0.417765127 | 6.40E-16 | postive |
| AXL | AC008808.1 | 0.413233733 | 1.41E-15 | postive |
| BCL2 | AC008808.1 | 0.451625605 | 1.21E-18 | postive |
| KLF9 | AC008808.1 | 0.552388556 | 8.60E-29 | postive |
| ZBP1 | AC116366.2 | 0.403604615 | 7.19E-15 | postive |
| CYLD | AC116366.2 | 0.54600916 | 4.81E-28 | postive |
| CFLAR | AC116366.2 | 0.516730731 | 8.27E-25 | postive |
| BCL2 | AC116366.2 | 0.432225187 | 4.80E-17 | postive |
| ATRX | AC116366.2 | 0.434979407 | 2.89E-17 | postive |
| KLF9 | DNAH10OS | 0.484116751 | 1.48E-21 | postive |
| CYLD | FAM13A-AS1 | 0.405405379 | 5.32E-15 | postive |
| KLF9 | AC015922.2 | 0.445743726 | 3.80E-18 | postive |
| FADD | AC004540.2 | 0.423341907 | 2.39E-16 | postive |
| DNMT1 | AC004540.2 | 0.649260079 | 1.94E-42 | postive |
| ATRX | AP000240.1 | 0.44166191 | 8.27E-18 | postive |
| KLF9 | PART1 | 0.576829382 | 8.22E-32 | postive |
| ATRX | AC007619.1 | 0.46655457 | 6.09E-20 | postive |
| BNIP3 | AC111182.1 | 0.417685579 | 6.49E-16 | postive |
| SPATA2 | AC124067.4 | 0.445439498 | 4.02E-18 | postive |
| DIABLO | AC004241.1 | 0.438560719 | 1.48E-17 | postive |
| AXL | FRMD6-AS2 | 0.444222837 | 5.08E-18 | postive |
| KLF9 | LINC01579 | 0.517846936 | 6.31E-25 | postive |
| CYLD | AC093423.2 | 0.463156925 | 1.22E-19 | postive |
| BCL2 | AC093423.2 | 0.499346923 | 4.96E-23 | postive |
| FLT3 | AC093423.2 | 0.492203329 | 2.49E-22 | postive |
| BACH2 | AC093423.2 | 0.449688382 | 1.77E-18 | postive |
| AXL | LINC01354 | 0.455762637 | 5.38E-19 | postive |
| BCL2 | LINC01354 | 0.512203248 | 2.46E-24 | postive |
| PLK1 | LINC01354 | -0.41603517 | 8.66E-16 | negative |
| KLF9 | LINC01354 | 0.73206068 | 8.41E-59 | postive |
| BCL2 | LINC00092 | 0.570298485 | 5.58E-31 | postive |
| FLT3 | LINC00092 | 0.489577748 | 4.47E-22 | postive |
| BACH2 | LINC00092 | 0.418147879 | 5.99E-16 | postive |
| KLF9 | LINC00092 | 0.47597736 | 8.53E-21 | postive |
| KLF9 | GNG12-AS1 | 0.649432104 | 1.82E-42 | postive |
| BCL2 | ZNF710-AS1 | 0.434787315 | 2.99E-17 | postive |
| IDH2 | ZNF710-AS1 | 0.503009164 | 2.13E-23 | postive |
| KLF9 | ZNF710-AS1 | 0.587308693 | 3.47E-33 | postive |
| FASLG | AC007991.4 | 0.579432673 | 3.79E-32 | postive |
| ZBP1 | AC007991.4 | 0.508969631 | 5.30E-24 | postive |
| TNFSF10 | AC007991.4 | 0.44947696 | 1.85E-18 | postive |
| AXL | BVES-AS1 | 0.408205719 | 3.32E-15 | postive |
| BCL2 | BVES-AS1 | 0.417905872 | 6.25E-16 | postive |
| KLF9 | BVES-AS1 | 0.644493993 | 1.19E-41 | postive |
| CYLD | AC145098.1 | 0.479794899 | 3.78E-21 | postive |
| BCL2 | AC145098.1 | 0.482489624 | 2.11E-21 | postive |
| FLT3 | AC145098.1 | 0.488557248 | 5.61E-22 | postive |
| BACH2 | AC145098.1 | 0.588204032 | 2.64E-33 | postive |
| HDAC9 | AC145098.1 | 0.486711167 | 8.42E-22 | postive |
| CYLD | LINC01857 | 0.420904865 | 3.69E-16 | postive |
| BCL2 | LINC01857 | 0.565225627 | 2.40E-30 | postive |
| FLT3 | LINC01857 | 0.512397359 | 2.35E-24 | postive |
| BACH2 | LINC01857 | 0.790484335 | 1.37E-74 | postive |
| AXL | CARMN | 0.48405459 | 1.50E-21 | postive |
| BCL2 | CARMN | 0.477892486 | 5.67E-21 | postive |
| KLF9 | CARMN | 0.758983418 | 1.62E-65 | postive |
| ATRX | AC002558.2 | 0.454109401 | 7.46E-19 | postive |
| CFLAR | AC087286.4 | 0.462578142 | 1.37E-19 | postive |
| CYLD | AL161725.1 | 0.586141345 | 4.97E-33 | postive |
| BCL2 | AL161725.1 | 0.554027761 | 5.49E-29 | postive |
| FLT3 | AL161725.1 | 0.562033118 | 5.93E-30 | postive |
| BACH2 | AL161725.1 | 0.572070938 | 3.33E-31 | postive |
| GATA3 | AC006007.1 | 0.420235144 | 4.15E-16 | postive |
| KLF9 | AC006007.1 | 0.596745728 | 1.82E-34 | postive |
| KLF9 | CACNA1C-AS1 | 0.441385515 | 8.71E-18 | postive |
| DIABLO | AL355490.2 | 0.42753373 | 1.13E-16 | postive |
| AXL | PGM5-AS1 | 0.481262477 | 2.75E-21 | postive |
| BCL2 | PGM5-AS1 | 0.443974637 | 5.33E-18 | postive |
| KLF9 | PGM5-AS1 | 0.713887261 | 1.03E-54 | postive |
| PLK1 | AC012073.1 | 0.493291339 | 1.96E-22 | postive |
| SPATA2 | AL034550.1 | 0.424414518 | 1.98E-16 | postive |
| AXL | AC010478.1 | 0.464677401 | 8.94E-20 | postive |
| BCL2 | AC010478.1 | 0.484135341 | 1.48E-21 | postive |
| KLF9 | AC010478.1 | 0.59459992 | 3.59E-34 | postive |
| CYLD | AC093495.1 | 0.402989211 | 7.97E-15 | postive |
| CYLD | AC008083.3 | 0.402328163 | 8.89E-15 | postive |
| BCL2 | AC008083.3 | 0.412423034 | 1.62E-15 | postive |
| BACH2 | AC008083.3 | 0.481020181 | 2.90E-21 | postive |
| HDAC9 | AC008083.3 | 0.544935394 | 6.41E-28 | postive |
| AXL | NR2F1-AS1 | 0.605954406 | 9.31E-36 | postive |
| BCL2 | NR2F1-AS1 | 0.440367036 | 1.06E-17 | postive |
| KLF9 | NR2F1-AS1 | 0.612565646 | 1.04E-36 | postive |
| BACH2 | AC011450.1 | 0.411158882 | 2.01E-15 | postive |
| DIABLO | AC127024.4 | -0.428875674 | 8.84E-17 | negative |
| ATRX | AC127024.4 | 0.480665469 | 3.13E-21 | postive |
| FADD | AL606489.1 | 0.413374987 | 1.37E-15 | postive |
| TRIM11 | AL606489.1 | 0.45172271 | 1.19E-18 | postive |
| FASLG | AL157871.2 | 0.539000019 | 3.06E-27 | postive |
| ZBP1 | AL157871.2 | 0.407998334 | 3.44E-15 | postive |
| KLF9 | AC087623.3 | 0.548730025 | 2.32E-28 | postive |
| CDKN2A | AC243964.3 | 0.417031876 | 7.28E-16 | postive |
| KLF9 | AC026691.1 | 0.465229078 | 7.99E-20 | postive |
| CYLD | LBX2-AS1 | -0.433596081 | 3.73E-17 | negative |
| BCL2 | LBX2-AS1 | -0.427221277 | 1.19E-16 | negative |
| CYLD | AC012181.1 | 0.428919724 | 8.77E-17 | postive |
| FADD | AP002784.1 | 0.474892509 | 1.07E-20 | postive |
| DNMT1 | AP002784.1 | 0.713971061 | 9.90E-55 | postive |
| TARDBP | AC008735.2 | 0.503890407 | 1.74E-23 | postive |
| CDKN2A | AC080129.2 | 0.435013479 | 2.87E-17 | postive |
| FASLG | AC007991.2 | 0.469874604 | 3.07E-20 | postive |
| ZBP1 | AC007991.2 | 0.47375827 | 1.36E-20 | postive |
| TNFSF10 | AC007991.2 | 0.419618671 | 4.62E-16 | postive |
| TARDBP | TMEM147-AS1 | 0.439130446 | 1.33E-17 | postive |
| CYLD | PXN-AS1 | -0.436504478 | 2.18E-17 | negative |
| DIABLO | PXN-AS1 | 0.409829223 | 2.52E-15 | postive |
| KLF9 | TPM1-AS | 0.594923754 | 3.24E-34 | postive |
| IPMK | AL049555.1 | 0.4112046 | 1.99E-15 | postive |
| HSP90AA1 | AC010976.1 | 0.40627681 | 4.60E-15 | postive |
| TARDBP | AC010976.1 | 0.545852893 | 5.02E-28 | postive |
| ATRX | BACH1-IT1 | 0.407063848 | 4.03E-15 | postive |
| FLT3 | AC103858.1 | 0.402792051 | 8.23E-15 | postive |
| DIABLO | OGFRP1 | 0.462012605 | 1.54E-19 | postive |
| STUB1 | AC114730.1 | 0.41434266 | 1.16E-15 | postive |
| BCL2 | Z97989.1 | 0.441348427 | 8.77E-18 | postive |
| KLF9 | Z97989.1 | 0.481623079 | 2.55E-21 | postive |
| CYLD | AL355076.2 | 0.420719121 | 3.81E-16 | postive |
| BCL2 | AL355076.2 | 0.529123727 | 3.86E-26 | postive |
| FLT3 | AL355076.2 | 0.487818941 | 6.60E-22 | postive |
| BACH2 | AL355076.2 | 0.529608163 | 3.42E-26 | postive |
| ATRX | AGBL5-IT1 | 0.471913948 | 2.01E-20 | postive |
| CYLD | FAM30A | 0.537352468 | 4.70E-27 | postive |
| BCL2 | FAM30A | 0.608403344 | 4.15E-36 | postive |
| FLT3 | FAM30A | 0.650610392 | 1.16E-42 | postive |
| BACH2 | FAM30A | 0.766212501 | 1.80E-67 | postive |
| ATRX | AC073569.1 | 0.506800328 | 8.82E-24 | postive |
| PLK1 | VPS9D1-AS1 | 0.405486321 | 5.25E-15 | postive |
| ATRX | AC011477.1 | 0.538016028 | 3.95E-27 | postive |
| KLF9 | AC007193.2 | 0.450315527 | 1.57E-18 | postive |
| CYLD | AL158207.2 | 0.446930149 | 3.02E-18 | postive |
| CFLAR | AL158207.2 | 0.445941831 | 3.66E-18 | postive |
| BCL2 | AL158207.2 | 0.486873501 | 8.13E-22 | postive |
| SIRT1 | AL158207.2 | 0.4012959 | 1.06E-14 | postive |
| BACH2 | AL158207.2 | 0.439353088 | 1.28E-17 | postive |
| RNF31 | AC068389.3 | -0.41291831 | 1.48E-15 | negative |
| FASLG | AC067945.2 | 0.521604556 | 2.52E-25 | postive |
| CYLD | AC067945.2 | 0.550634628 | 1.39E-28 | postive |
| FLT3 | AC067945.2 | 0.462059349 | 1.52E-19 | postive |
| CD40 | AC067945.2 | 0.43023426 | 6.91E-17 | postive |
| BACH2 | AC006058.1 | 0.518341699 | 5.59E-25 | postive |
| CYLD | LRRC8C-DT | 0.500735057 | 3.61E-23 | postive |
| AXL | LRRC8C-DT | 0.467676803 | 4.83E-20 | postive |
| BCL2 | LRRC8C-DT | 0.625878968 | 1.06E-38 | postive |
| FLT3 | LRRC8C-DT | 0.564586286 | 2.88E-30 | postive |
| BACH2 | LRRC8C-DT | 0.492420395 | 2.38E-22 | postive |
| KLF9 | LRRC8C-DT | 0.403221134 | 7.67E-15 | postive |
| BCL2 | AC119396.2 | 0.407699684 | 3.62E-15 | postive |
| BACH2 | AC119396.2 | 0.513406873 | 1.84E-24 | postive |
| KLF9 | AC092691.1 | 0.505560092 | 1.18E-23 | postive |
| DIABLO | PSMA3-AS1 | -0.406010457 | 4.81E-15 | negative |
| STUB1 | PSMA3-AS1 | -0.418477326 | 5.65E-16 | negative |
| ATRX | PSMA3-AS1 | 0.527702061 | 5.52E-26 | postive |
| FASLG | AC004988.1 | 0.471121393 | 2.37E-20 | postive |
| ALK | SOX2-OT | 0.436853155 | 2.04E-17 | postive |
| TARDBP | AC005519.1 | 0.43399591 | 3.46E-17 | postive |
| KLF9 | AC005906.2 | 0.501327092 | 3.15E-23 | postive |
| AXL | LINC02106 | 0.465747547 | 7.19E-20 | postive |
| BCL2 | LINC02106 | 0.441587754 | 8.38E-18 | postive |
| KLF9 | LINC02106 | 0.654218537 | 2.84E-43 | postive |
| DIABLO | AC019080.3 | -0.423921934 | 2.16E-16 | negative |
| ATRX | AC019080.3 | 0.403594262 | 7.20E-15 | postive |
| AXL | AC245041.2 | 0.462384137 | 1.43E-19 | postive |
| CYLD | ANKRD44-IT1 | 0.437431514 | 1.83E-17 | postive |
| BCL2 | ANKRD44-IT1 | 0.597542635 | 1.41E-34 | postive |
| FLT3 | ANKRD44-IT1 | 0.430768358 | 6.27E-17 | postive |
| BACH2 | ANKRD44-IT1 | 0.641370046 | 3.86E-41 | postive |
| TRIM11 | MAGI2-AS3 | -0.49655589 | 9.37E-23 | negative |
| CYLD | MAGI2-AS3 | 0.44275234 | 6.72E-18 | postive |
| AXL | MAGI2-AS3 | 0.531304702 | 2.22E-26 | postive |
| BCL2 | MAGI2-AS3 | 0.523908698 | 1.42E-25 | postive |
| PLK1 | MAGI2-AS3 | -0.50478468 | 1.41E-23 | negative |
| IDH1 | MAGI2-AS3 | -0.400447032 | 1.21E-14 | negative |
| KLF9 | MAGI2-AS3 | 0.744081307 | 1.07E-61 | postive |
| BCL2 | AL590609.2 | 0.50293222 | 2.17E-23 | postive |
| BACH2 | AL590609.2 | 0.534892396 | 8.87E-27 | postive |
| AXL | SCAT1 | 0.458717059 | 2.99E-19 | postive |
| ALK | AC005725.1 | 0.46878233 | 3.85E-20 | postive |
| TSC1 | AC015813.1 | 0.401765825 | 9.76E-15 | postive |
| HSP90AA1 | RUSC1-AS1 | 0.42555387 | 1.61E-16 | postive |
| TARDBP | RUSC1-AS1 | 0.502211295 | 2.57E-23 | postive |
| DIABLO | AC005562.1 | 0.481331084 | 2.71E-21 | postive |
| KLF9 | AC012085.2 | 0.55750336 | 2.10E-29 | postive |
| MAP3K7 | AL359715.2 | 0.411771409 | 1.81E-15 | postive |
| CYLD | AL359220.1 | 0.441982453 | 7.78E-18 | postive |
| CFLAR | AL359220.1 | 0.401328319 | 1.05E-14 | postive |
| BCL2 | AL359220.1 | 0.446252159 | 3.44E-18 | postive |
| FLT3 | AL359220.1 | 0.471886132 | 2.02E-20 | postive |
| BACH2 | AL359220.1 | 0.400178616 | 1.27E-14 | postive |
| TARDBP | SNHG1 | 0.545400911 | 5.66E-28 | postive |
| TSC1 | AC007066.2 | 0.493059462 | 2.06E-22 | postive |
| TARDBP | AC007066.2 | 0.441727277 | 8.16E-18 | postive |
| BCL2 | CADM3-AS1 | 0.50912804 | 5.10E-24 | postive |
| FLT3 | CADM3-AS1 | 0.460106461 | 2.26E-19 | postive |
| KLF9 | CADM3-AS1 | 0.620540764 | 6.84E-38 | postive |
| OTULIN | COX10-AS1 | 0.427584744 | 1.12E-16 | postive |
| MAP3K7 | AL359715.1 | 0.428846259 | 8.89E-17 | postive |
| IDH2 | LINC01578 | 0.638244406 | 1.23E-40 | postive |
| BCL2 | SNHG14 | 0.40031494 | 1.24E-14 | postive |
| KLF9 | SNHG14 | 0.590042583 | 1.49E-33 | postive |
| FAS | LINC02528 | 0.418006601 | 6.14E-16 | postive |
| FASLG | LINC02528 | 0.697094962 | 3.30E-51 | postive |
| ZBP1 | LINC02528 | 0.405535609 | 5.21E-15 | postive |
| TNFSF10 | LINC02528 | 0.414915139 | 1.05E-15 | postive |
| CD40 | LINC02528 | 0.411201772 | 1.99E-15 | postive |
| BNIP3 | AC092969.1 | 0.404462881 | 6.23E-15 | postive |
| DIABLO | MALAT1 | -0.564026912 | 3.37E-30 | negative |
| ATRX | MALAT1 | 0.608845031 | 3.59E-36 | postive |
| HDAC9 | AL356417.1 | 0.621606883 | 4.73E-38 | postive |
| KLF9 | AC037198.2 | 0.577409405 | 6.92E-32 | postive |
| BCL2 | AL161457.1 | 0.459722065 | 2.44E-19 | postive |
| KLF9 | AL161457.1 | 0.708885813 | 1.21E-53 | postive |
| AXL | AC009806.1 | 0.417563699 | 6.63E-16 | postive |
| KLF9 | AC009806.1 | 0.556801626 | 2.56E-29 | postive |
| KLF9 | AC079313.2 | 0.438981696 | 1.37E-17 | postive |
| ATRX | AC005776.2 | 0.480500665 | 3.25E-21 | postive |
| ZBP1 | AC008105.3 | 0.470430954 | 2.73E-20 | postive |
| CYLD | AC008105.3 | 0.581983138 | 1.76E-32 | postive |
| CFLAR | AC008105.3 | 0.483920436 | 1.55E-21 | postive |
| BCL2 | AC008105.3 | 0.526127241 | 8.19E-26 | postive |
| FLT3 | AC008105.3 | 0.583454395 | 1.13E-32 | postive |
| BACH2 | AC008105.3 | 0.593072094 | 5.80E-34 | postive |
| CD40 | AC008105.3 | 0.409044511 | 2.88E-15 | postive |
| FADD | AL355312.3 | 0.42279952 | 2.63E-16 | postive |
| RNF31 | AL355312.3 | 0.422184049 | 2.94E-16 | postive |
| CYLD | AL024508.1 | -0.427742321 | 1.09E-16 | negative |
| AXL | BARX1-DT | 0.471478713 | 2.20E-20 | postive |
| BCL2 | BARX1-DT | 0.421932382 | 3.07E-16 | postive |
| KLF9 | BARX1-DT | 0.768145474 | 5.25E-68 | postive |
| HDAC9 | LINC02202 | 0.401317846 | 1.05E-14 | postive |
| AXL | AC010980.2 | 0.402245024 | 9.02E-15 | postive |
| KLF9 | AC010980.2 | 0.65477268 | 2.29E-43 | postive |
| CYLD | SUCLA2-AS1 | -0.423985835 | 2.13E-16 | negative |
| CFLAR | SUCLA2-AS1 | -0.403900594 | 6.85E-15 | negative |
| FADD | AP000487.2 | 0.416313184 | 8.25E-16 | postive |
| CYLD | AC008569.2 | 0.424916069 | 1.81E-16 | postive |
| BCL2 | AC008569.2 | 0.415791845 | 9.03E-16 | postive |
| BACH2 | AC008569.2 | 0.427285864 | 1.18E-16 | postive |
| ATRX | AC008569.2 | 0.50882741 | 5.48E-24 | postive |
| AXL | RBMS3-AS3 | 0.460738671 | 1.99E-19 | postive |
| BCL2 | RBMS3-AS3 | 0.438023406 | 1.64E-17 | postive |
| PLK1 | RBMS3-AS3 | -0.415259196 | 9.90E-16 | negative |
| KLF9 | RBMS3-AS3 | 0.611921187 | 1.29E-36 | postive |
| ZBP1 | AL023653.1 | 0.461660868 | 1.65E-19 | postive |
| CYLD | AL023653.1 | 0.51678371 | 8.17E-25 | postive |
| BCL2 | AL023653.1 | 0.521665971 | 2.48E-25 | postive |
| FLT3 | AL023653.1 | 0.504851109 | 1.39E-23 | postive |
| BACH2 | AL023653.1 | 0.650109198 | 1.40E-42 | postive |
| DNMT1 | MAFA-AS1 | 0.586156147 | 4.95E-33 | postive |
| AXL | AP001107.5 | 0.496384513 | 9.74E-23 | postive |
| BCL2 | AP001107.5 | 0.449284412 | 1.92E-18 | postive |
| KLF9 | AP001107.5 | 0.749013665 | 6.26E-63 | postive |
| AXL | NKILA | 0.451842846 | 1.16E-18 | postive |
| KLF9 | FLRT1 | 0.619358283 | 1.03E-37 | postive |
| DIABLO | AC016949.1 | -0.406907525 | 4.13E-15 | negative |
| ATRX | AC016949.1 | 0.4238517 | 2.18E-16 | postive |
| ATRX | FOXP1-AS1 | 0.408229521 | 3.31E-15 | postive |
| TARDBP | AC079807.1 | -0.411673792 | 1.84E-15 | negative |
| ATRX | AC011472.4 | 0.443742534 | 5.57E-18 | postive |
| KLF9 | AC011472.4 | 0.627897049 | 5.20E-39 | postive |
| KLF9 | LINC00702 | 0.421431187 | 3.36E-16 | postive |
| FADD | AP002336.2 | 0.533393046 | 1.30E-26 | postive |
| HDAC9 | LINC01679 | 0.502244442 | 2.55E-23 | postive |
| FAS | HLA-DQB1-AS1 | 0.40593669 | 4.87E-15 | postive |
| FASLG | HLA-DQB1-AS1 | 0.404201345 | 6.51E-15 | postive |
| CYLD | HLA-DQB1-AS1 | 0.529257295 | 3.73E-26 | postive |
| FLT3 | HLA-DQB1-AS1 | 0.439336069 | 1.28E-17 | postive |
| BACH2 | HLA-DQB1-AS1 | 0.473206761 | 1.53E-20 | postive |
| CD40 | HLA-DQB1-AS1 | 0.429327193 | 8.15E-17 | postive |
| AXL | AC015660.2 | 0.457427381 | 3.86E-19 | postive |
| TRIM11 | MIR99AHG | -0.405366834 | 5.36E-15 | negative |
| AXL | MIR99AHG | 0.42169543 | 3.20E-16 | postive |
| BCL2 | MIR99AHG | 0.469777397 | 3.13E-20 | postive |
| PLK1 | MIR99AHG | -0.511288358 | 3.06E-24 | negative |
| KLF9 | MIR99AHG | 0.587215067 | 3.58E-33 | postive |
| TERT | POU6F2-AS1 | 0.403338701 | 7.52E-15 | postive |
| KLF9 | AC015845.2 | 0.569821102 | 6.41E-31 | postive |
| KLF9 | AP001189.3 | 0.417741474 | 6.43E-16 | postive |
| AXL | AC067750.1 | 0.43989781 | 1.15E-17 | postive |
| BCL2 | AC067750.1 | 0.404795907 | 5.89E-15 | postive |
| KLF9 | AC067750.1 | 0.697045128 | 3.38E-51 | postive |
| IDH2 | AL731566.2 | 0.723652205 | 7.20E-57 | postive |
| TARDBP | AL135999.1 | 0.455575551 | 5.58E-19 | postive |
| KLF9 | FGF14-AS2 | 0.506559459 | 9.34E-24 | postive |
| AXL | MBNL1-AS1 | 0.438376494 | 1.53E-17 | postive |
| BCL2 | MBNL1-AS1 | 0.401497305 | 1.02E-14 | postive |
| KLF9 | MBNL1-AS1 | 0.606402787 | 8.03E-36 | postive |
| BACH2 | AL445423.1 | 0.418618871 | 5.51E-16 | postive |
| KLF9 | AC145124.1 | 0.460970687 | 1.90E-19 | postive |
| DIABLO | AC007036.2 | -0.454564786 | 6.82E-19 | negative |
| ATRX | AL138787.2 | 0.469107031 | 3.60E-20 | postive |
| BCL2 | LINC00476 | 0.403861897 | 6.89E-15 | postive |
| BCL2 | LINC01197 | 0.56254631 | 5.13E-30 | postive |
| FLT3 | LINC01197 | 0.513339782 | 1.87E-24 | postive |
| KLF9 | LINC01197 | 0.490483894 | 3.66E-22 | postive |
| CYLD | CHRM3-AS2 | 0.513241535 | 1.92E-24 | postive |
| BCL2 | CHRM3-AS2 | 0.466073681 | 6.72E-20 | postive |
| FLT3 | CHRM3-AS2 | 0.543926563 | 8.38E-28 | postive |
| BACH2 | CHRM3-AS2 | 0.62157794 | 4.78E-38 | postive |
| LEF1 | CHRM3-AS2 | 0.448013719 | 2.45E-18 | postive |
| CYLD | FOXP4-AS1 | -0.437444688 | 1.83E-17 | negative |
| BNIP3 | LINC01829 | 0.45683301 | 4.35E-19 | postive |
| FADD | LINC02584 | 0.509917023 | 4.23E-24 | postive |
| BCL2 | AC133065.1 | 0.441446781 | 8.61E-18 | postive |
| BACH2 | AC133065.1 | 0.439638095 | 1.21E-17 | postive |
| HDAC9 | AC133065.1 | 0.546077161 | 4.73E-28 | postive |
| FASLG | AC022126.1 | 0.519177844 | 4.56E-25 | postive |
| TNFSF10 | AC022126.1 | 0.417035985 | 7.27E-16 | postive |
| FASLG | MMP25-AS1 | 0.474253026 | 1.23E-20 | postive |
| BNIP3 | MIR9-3HG | 0.402733293 | 8.32E-15 | postive |
| APP | FGF13-AS1 | 0.444698435 | 4.64E-18 | postive |
| IDH2 | AC087284.1 | 0.517247682 | 7.30E-25 | postive |
| STUB1 | AC092720.1 | 0.476101799 | 8.30E-21 | postive |
| RNF31 | AC092720.1 | 0.423882679 | 2.17E-16 | postive |
| DIABLO | AC015849.3 | -0.500247613 | 4.03E-23 | negative |
| ATRX | AC015849.3 | 0.496430853 | 9.63E-23 | postive |
| AXL | LINC02657 | 0.649663357 | 1.67E-42 | postive |
| DIABLO | AC103769.1 | -0.405624852 | 5.13E-15 | negative |
| SPATA2 | GASAL1 | 0.41657758 | 7.88E-16 | postive |
| DIABLO | AL133245.1 | -0.435400463 | 2.67E-17 | negative |
| ATRX | AL133245.1 | 0.537363361 | 4.69E-27 | postive |
| KLF9 | LINC01797 | 0.499536508 | 4.75E-23 | postive |
| BCL2 | AC069549.1 | 0.430748383 | 6.29E-17 | postive |
| DIABLO | AC005086.2 | -0.411629709 | 1.85E-15 | negative |
| FADD | AP003555.2 | 0.541908033 | 1.43E-27 | postive |
| BCL2 | ZNF582-AS1 | 0.43089768 | 6.12E-17 | postive |
| KLF9 | ZNF582-AS1 | 0.527544846 | 5.74E-26 | postive |
| BCL2 | AL137779.1 | 0.44773889 | 2.59E-18 | postive |
| BACH2 | AL137779.1 | 0.5392962 | 2.83E-27 | postive |
| BCL2 | AC124312.3 | 0.442196572 | 7.47E-18 | postive |
| KLF9 | AC124312.3 | 0.674860992 | 6.32E-47 | postive |
| KLF9 | LRRC3-DT | 0.683305299 | 1.66E-48 | postive |
| CYLD | AC108860.2 | -0.458818451 | 2.93E-19 | negative |
| HDAC9 | AC107884.1 | 0.507300793 | 7.85E-24 | postive |
| AXL | AL391121.1 | 0.486803868 | 8.25E-22 | postive |
| HAT1 | AL391121.1 | -0.405373157 | 5.35E-15 | negative |
| TARDBP | AL391121.1 | -0.534577253 | 9.62E-27 | negative |
| HDAC9 | AC017104.1 | 0.579592473 | 3.61E-32 | postive |
| BCL2 | LINC01678 | 0.411183637 | 2.00E-15 | postive |
| FLT3 | LINC01678 | 0.403386391 | 7.46E-15 | postive |
| DNMT1 | AC025280.2 | 0.414753059 | 1.08E-15 | postive |
| CFLAR | AP000759.1 | 0.412090159 | 1.71E-15 | postive |
| FASLG | AC026369.3 | 0.456218796 | 4.91E-19 | postive |
| PLK1 | AC093249.2 | 0.449218305 | 1.94E-18 | postive |
| TERT | LINC01619 | 0.601552173 | 3.90E-35 | postive |
| HDAC9 | AC023590.1 | 0.567019501 | 1.44E-30 | postive |
| HDAC9 | LINC02416 | 0.460558394 | 2.06E-19 | postive |
| BCL2 | AP001056.1 | 0.44038142 | 1.05E-17 | postive |
| FLT3 | AP001056.1 | 0.509294071 | 4.91E-24 | postive |
| BACH2 | AP001056.1 | 0.486627339 | 8.58E-22 | postive |
| ATRX | CCDC18-AS1 | 0.470045672 | 2.96E-20 | postive |
