# Supplementary material for: Necroptosis-Related lncRNAs: Predicting Prognosis and the Distinction between the Cold and Hot Tumors in Gastric Cancer
Source: J Oncol. 2021 Nov 8;2021:6718443. doi: 10.1155/2021/6718443 (PMC8592775; doi:10.1155/2021/6718443)
Supplement: Supplementary Materials — Appendix S1. Figure S1: the GSEA of the high-risk group and cluster 2. (A) The GSEA of the high-risk group. (B) The GSEA of cluster 2. Appendix S2. Figure S2: the IC50 prediction of 16 chemical or targeted drugs in risk groups. Appendix S3. Figure S3: consensus clustering analysis of necroptosis-related lncRNAs and IC50 prediction in clusters. (A) The heat map, cumulative distribution function (CDF) plot, and the consensus CDF plots of consensus clustering matrix. (B) 16 chemical or targeted drugs solely showing significant IC50 difference in clusters. Appendix T1: the table of necroptosis-related genes refers to GSEA and previous reports. Appendix D1: the network data of necroptosis-related genes and lncRNAs. Appendix D2: the profile of significantly differently infiltrated immune cells between risk groups in different platforms. Appendix D3: the profile of significantly differently infiltrated immune cells between clusters in different platforms. [file 6718443.f1.zip › 6718443.f1/Appendix D3.docx]

| immune | pvalue |
| --- | --- |
| B cell_TIMER | 0.000482509 |
| T cell CD4+_TIMER | 0.008355563 |
| T cell CD8+_TIMER | 0.001684616 |
| Neutrophil_TIMER | 0.000194565 |
| Macrophage_TIMER | 0.001438125 |
| Myeloid dendritic cell_TIMER | 3.41E-06 |
| B cell memory_CIBERSORT | 2.80E-06 |
| T cell CD8+_CIBERSORT | 8.91E-06 |
| NK cell resting_CIBERSORT | 0.000764001 |
| NK cell activated_CIBERSORT | 0.033284167 |
| Macrophage M0_CIBERSORT | 4.93E-05 |
| Macrophage M2_CIBERSORT | 0.006480975 |
| Mast cell activated_CIBERSORT | 0.000173039 |
| Mast cell resting_CIBERSORT | 0.000888214 |
| B cell memory_CIBERSORT-ABS | 1.96E-06 |
| T cell CD8+_CIBERSORT-ABS | 1.22E-06 |
| T cell regulatory (Tregs)_CIBERSORT-ABS | 0.020707932 |
| NK cell resting_CIBERSORT-ABS | 0.002042644 |
| NK cell activated_CIBERSORT-ABS | 0.001353131 |
| Monocyte_CIBERSORT-ABS | 0.006880111 |
| Macrophage M0_CIBERSORT-ABS | 0.00778264 |
| Macrophage M1_CIBERSORT-ABS | 0.014006709 |
| Macrophage M2_CIBERSORT-ABS | 4.74E-05 |
| Mast cell activated_CIBERSORT-ABS | 8.42E-05 |
| Mast cell resting_CIBERSORT-ABS | 0.010264926 |
| B cell_QUANTISEQ | 1.63E-06 |
| Macrophage M2_QUANTISEQ | 0.000414867 |
| Neutrophil_QUANTISEQ | 0.018477291 |
| T cell CD8+_QUANTISEQ | 0.001866833 |
| T cell regulatory (Tregs)_QUANTISEQ | 0.002484909 |
| uncharacterized cell_QUANTISEQ | 0.00098799 |
| T cell_MCPCOUNTER | 0.033273991 |
| cytotoxicity score_MCPCOUNTER | 0.00102447 |
| NK cell_MCPCOUNTER | 0.003541986 |
| B cell_MCPCOUNTER | 3.28E-05 |
| Monocyte_MCPCOUNTER | 0.002060963 |
| Macrophage/Monocyte_MCPCOUNTER | 0.002060963 |
| Myeloid dendritic cell_MCPCOUNTER | 8.29E-08 |
| Endothelial cell_MCPCOUNTER | 0.029021633 |
| Cancer associated fibroblast_MCPCOUNTER | 0.000618889 |
| Myeloid dendritic cell activated_XCELL | 1.57E-08 |
| B cell_XCELL | 0.000336224 |
| T cell CD4+ naive_XCELL | 0.000674904 |
| T cell CD4+ (non-regulatory)_XCELL | 0.003884752 |
| T cell CD8+_XCELL | 2.85E-06 |
| T cell CD8+ central memory_XCELL | 1.37E-07 |
| T cell CD8+ effector memory_XCELL | 0.020963391 |
| Class-switched memory B cell_XCELL | 0.000136973 |
| Common lymphoid progenitor_XCELL | 0.016198552 |
| Myeloid dendritic cell_XCELL | 7.58E-06 |
| Endothelial cell_XCELL | 0.003407427 |
| Eosinophil_XCELL | 0.032469375 |
| Cancer associated fibroblast_XCELL | 6.89E-07 |
| Granulocyte-monocyte progenitor_XCELL | 0.008453481 |
| Hematopoietic stem cell_XCELL | 2.14E-05 |
| Macrophage_XCELL | 0.005993707 |
| Macrophage M1_XCELL | 0.012434284 |
| Mast cell_XCELL | 0.005124563 |
| B cell memory_XCELL | 0.003239935 |
| immune score_XCELL | 4.54E-06 |
| stroma score_XCELL | 4.96E-06 |
| microenvironment score_XCELL | 1.40E-08 |
| B cell_EPIC | 5.18E-05 |
| T cell CD4+_EPIC | 0.008482189 |
| Endothelial cell_EPIC | 0.004636481 |
| Macrophage_EPIC | 0.000264571 |
| uncharacterized cell_EPIC | 1.07E-05 |
